# A critical survey of vestigial structures in the postcranial skeletons of extant mammals

**DOI:** 10.7717/peerj.1439

**Published:** 2015-11-24

**Authors:** Phil Senter, John G. Moch

**Affiliations:** 1 Department of Biological Sciences, Fayetteville State University, Fayetteville, NC, United States; 2 Department of Chemistry and Physics, Fayetteville State University, Fayetteville, NC, United States

**Keywords:** Vestigial structures, Vestigial organs, Evolution, Mammalia, Osteology, Anatomy

## Abstract

In the Mammalia, vestigial skeletal structures abound but have not previously been the focus of study, with a few exceptions (e.g., whale pelves). Here we use a phylogenetic bracketing approach to identify vestigial structures in mammalian postcranial skeletons and present a descriptive survey of such structures in the Mammalia. We also correct previous misidentifications, including the previous misidentification of vestigial caviid metatarsals as sesamoids. We also examine the phylogenetic distribution of vestigiality and loss. This distribution indicates multiple vestigialization and loss events in mammalian skeletal structures, especially in the hand and foot, and reveals no correlation in such events between mammalian fore and hind limbs.

## Introduction

A vestigial structure is a biological structure that has lost a major ancestral function and is usually drastically reduced in size. Well-known examples include the eyes of blind cave fishes and blind cave salamanders, and the diminutive wings of kiwis and emus. As early as the eighteenth century, Erasmus Darwin (1791) recognized vestigial structures as evidence for biological evolution, and such recognition continues among today’s biologists and paleontologists (e.g., Prothero, 2007; Hall & Hallgrimsson, 2008; Senter et al., 2015). For such structures Lamarck (1809) used the French words *rudiments* and *vestiges*. Charles Darwin (1859) used the term “rudimentary organs.” Wiedersheim (1895) popularized the use of the term “vestigial” for such structures, and such use continues today.

The term “vestigial” does not imply a complete lack of any function. Although some biologists maintain that it does (e.g., Prothero, 2007; Bergstrom & Dugatkin, 2012), most reject that strict view and follow Darwin (1859) in accepting that a vestigial structure has lost a salient function but may retain some other function (e.g., Bejder & Hall, 2002; Kearney, 2002; Hall, 2003; Simões-Lopes & Gutstein, 2004; Regoes et al., 2005; Espinasa & Jeffery, 2006; Franz-Odendaal & Hall, 2006; Prince & Johnson, 2006; Hall & Hallgrimsson, 2008; Zubidat, Nelson & Haim, 2010; Moch & Senter, 2011; Jackson & Auer, 2012). For example, the vestigial second and fourth metacarpals and metatarsals of horses no longer function as struts between a digit and the carpus or tarsus but still function as guides for suspensory ligaments and as muscle attachment sites, as well as supports for carpal and tarsal bones (Smythe, 1967; Jackson & Auer, 2012). Likewise, vestigial whale pelves have lost their ancestral locomotor function but still anchor muscles associated with the reproductive system (Struthers, 1881; Simões-Lopes & Gutstein, 2004).

**Figure 1:**
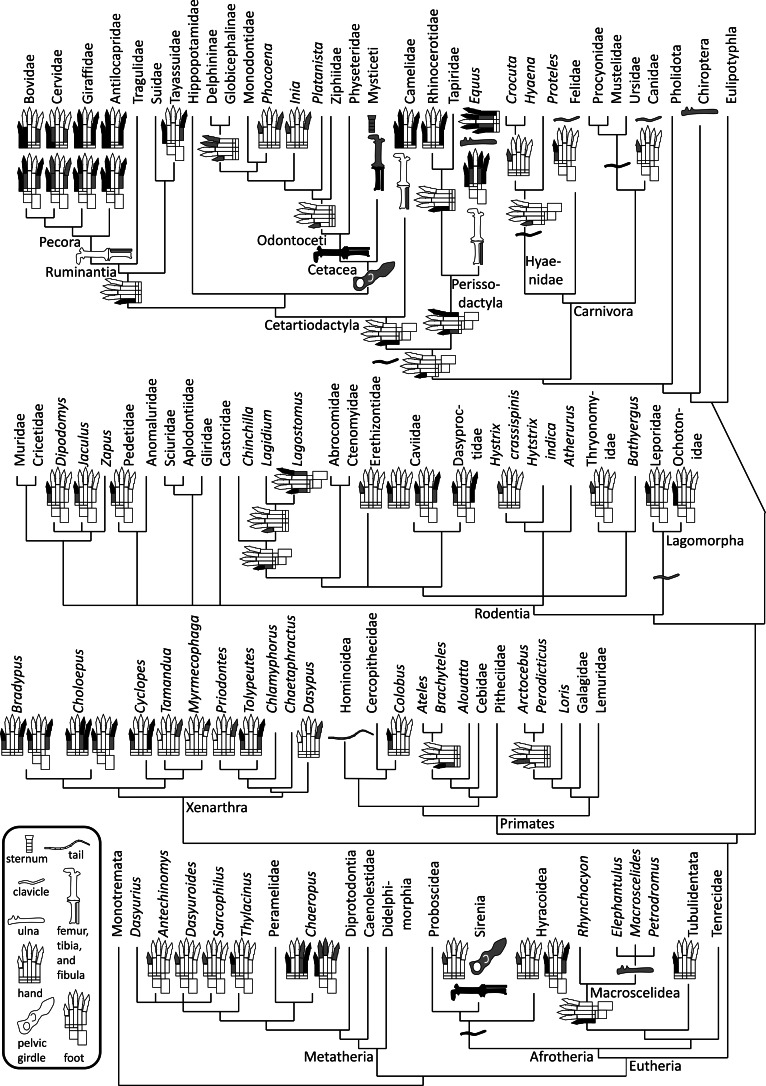
Phylogeny of bone vestigialization and loss in mammals. Phylogeny of extant Mammalia, showing phylogenetic distribution of vestigial (gray) and lost (black) skeletal structures. Gray ulna symbols refer to vestigiality of the ulnar shaft, not the entire ulna. Here, the phylogeny of the major mammalian clades is per Murphy et al. (2001). Phylogeny within Rodentia is a consensus of the studies of DeBry & Sagel (2001), Huchon & Douzery (2001), Montgelard et al. (2002) and Adkins, Walton & Honeycutt (2003). For phylogenies within other mammalian sub-clades we used the following sources. Afrotheria: Murata et al. (2003). Carnivora: Eizirik et al. (2010). Cetartiodactyla: Agnarsson & May-Collado (2008). Metatheria: Amrine-Madsen et al. (2003). Perissodactyla: Tougard et al. (2001). Primates: Fabre, Rodrigues & Douzery (2009). Xenarthra: Delsuc et al. (2012).

Vestigial structures are common in the postcranial skeletons of extant mammals (Fig. 1). The vestigial tails of humans, pelves of whales, and metacarpals and metatarsals of horses are frequently cited examples (e.g., Prothero, 2007; Kardong, 2008; Hall & Hallgrimsson, 2008). Many more examples exist, but most are little-known, and some have not previously been explicitly identified as vestigial. It would be useful to publish an illustrated survey of the vestigial structures in mammal postcrania and to trace the evolutionary trends in vestigiality and loss of postcranial skeletal structures across the Mammalia. We conducted this study so as to produce such a publication by answering three questions. First, for any given postcranial skeletal element, in which mammalian taxa is it vestigial? Second, for any given postcranial skeletal element, how many times (and in what taxa) has vestigialization and/or loss independently occurred in the Mammalia? Third, have any vestigial postcranial skeletal elements in the Mammalia previously been misidentified as something else? To increase the utility of the study for the non-specialist, we have included common names in American English along with taxonomic names in the main text.

## Materials and Methods

### Specimen examination

We examined 578 mammalian skeletal specimens and skins from 293 species (Table 1) representing 98 (70%) of the 139 extant mammal families listed by Nowak (1999). We supplemented our observations with previously published descriptions as needed. We also examined manual and pedal morphology in 74 live members of 23 hoofed species of the Cetartiodactyla and in museum-supplied photos of seven skeletal specimens of rhinoceroses and manatees (Table 1).

**Table 1:** List of examined specimens. Specimens examined for this study. Asterisks indicate skins; all other listed specimens are skeletal except where noted. See text for literature used in addition to specimen observations.

Order and family	Genus and species	Specimen numbers (where known)
**Order Monotremata**		
Ornithorhynchidae	*Ornithorhynchus anatinus*	AMNH 201266; USNM (NAN)
Tachyglossidae	*Tachyglossus aculeatus*	USNM (NAN)
**Clade Metatheria**		
**Order Dasyuromorphia**		
Dasyuridae	*Antechinus godmani*	USNM 23481
	*Dasyuroides byrnei*	USNM 396649, 464997
	*Dasyurus albopunctatus*	USNM 521036
	*D. geoffroyi*	USNM 237742
	*D. hallucatus*	USNM 283979
	*D. maculatus*	USNM (NAN)
	*Sarcophilus harrisii*	USNM 307639
	*S. laniarius*	USNM 8665, 173904
Thylacinidae	*Thylacinus cynocephalus*	USNM 49724, 155387, 238801
**Order Didelphimorphia**		
Didelphidae	*Didelphis marsupialis*	AMNH M40059
	*D. virginiana*	USNM (NAN)
**Order Diprotodontia**		
Macropodidae	*Dendrolagus lumholtzi*	AMNH M38425
	*Macropus canguru*	USNM (NAN)
	*M. eugenii*	NCSM 1283*, 1284*, 15212*, 15213*
	*M. rufus*	Three live animals
Phalangeridae	*Trichosurus vulpecula*	USNM (NAN)
Phascolarctidae	*Phascolarctos cinereus*	AMNH M242; USNM (NAN)
Potoroidae	*Aepyprymnus rufescens*	USNM 49738
	*Bettongia penicillata*	USNM 237719, 237720*, 237725
Pseudocheiridae	*Petauroides volans*	USNM (NAN)
**Order Peramelemorphia**		
Peramelidae	*Echimipera* sp.	USNM 595488
	*Isoodon macrourus*	USNM 237732, 284018
	*I. obesus*	USNM 237731
	*Perameles nasuta*	USNM 221208
Thylacomyidae	*Macrotis lagotis*	USNM A22990
**Clade Eutheria**		
**Order Afrosoricida**		
Chrysochloridae	*Chrysospalax trevelyani*	AMNH 34880
Tenrecidae	*Tenrec ecaudatus*	USNM (NAN)
**Order Carnivora**		
Canidae	*Canis aureus*	USNM (NAN)
	*C. familiaris*	AMNH (NAN), 80145, 204030; USNM (NAN)
	*C. latrans*	NCSM 2450, 5281, 7117, 8326, 8577, 8963, 13373
	*C. lupus*	AMNH 10417; NCSM 5284
	*Vulpes vulpes*	NCSM 15485; USNM (NAN)
Felidae	*Acinonyx jubatus*	USNM (NAN)
	*Caracal caracal*	USNM (NAN)
	*Felis catus*	FSU (NAN: six specimens); NCSM (NAN)
	*Leopardus pardalis*	AMNH 4; CTR (NAN)
	*Lynx rufus*	NCSM 15020
	*Panthera tigris*	USNM (NAN); CTR (NAN)
Herpestidae	*Herpestes javanicus*	NCSM 13402*, 13405*
Hyaenidae	*Crocuta crocuta*	AMNH 5142, 147880
	*Hyaena brunnea*	USNM 267891; USNM (NAN)
	*H. hyaena*	USNM 328576
	*Proteles cristatus*	USNM (NAN)
Mephitidae	*Mephitis mephitis*	USNM (NAN)
	*Spilogale gracilis*	USNM (NAN)
Mustelidae	*Eira barbara*	CTR (NAN)
	*Enhydra lutra*	USNM (NAN)
	*Lutra canadensis*	USNM (NAN)
	*Mustela erminea*	USNM (NAN), 16458, 498822*
	*M. nivalis*	NCSM 7838*; USNM 115211
Nandiniidae	*Nandinia binotata*	NCSM 8196*
Phocidae	*Phoca groenlandica*	USNM (NAN)
	*Ph. vitulina*	NCSM 384
Procyonidae	*Nasua nasua*	NCSM 13405*; USNM (NAN)
	*Procyon lotor*	NCSM 2446, 4000*, 4010*, 15030; USNM (NAN)
Ursidae	*Helarctos malayanus*	USNM (NAN)
	*Ursus americanus*	NCSM 6655*, 8381*
	*U. arctos*	USNM (NAN)
Viverridae	*Arctictis binturong*	CTR (NAN); NCSM 15483*
	*Viverra tangalunga*	USNM (NAN)
**Clade Cetartiodactyla**		
Antilocapridae	*Antilocapra americana*	USNM 256452, 259010; USNM (NAN)
Balaenidae	*Eubalaena glacialis*	NCSM 3286
Balaenopteridae	*Balaenoptera musculus*	NCSM 8381
	*Megaptera novaeangliae*	NCSM 8201
Bovidae	*Addax nasomaculatus*	One live animal
	*Ammotragus lervia*	NCSM 7674; Five live animals
	*Antilope cervicapra*	Two live animals
	*Bison bison*	NCSM 7673; Three live animals
	*Bos taurus*	NCSM 000356; Six live animals
	*Bubalus bubalus*	Five live animals
	*Hippotragus niger*	Three live animals
	*Nanger dama*	Two live animals
	*Oryx gazelle*	One live animal
	*Ovis canadensis*	Two live animals
	*O. aries*	Three live animals
	*Tragelaphus eurycerus*	One live animal
Camelidae	*Camelus dromedarius*	USNM (NAN); three live animals
	*C. ferus*	Four live animals
	*Lama glama*	USNM (NAN); five live animals
Cervidae	*Alces alces*	One live animal
	*Axis axis*	NCSM 14258; seven live animals
	*Cervus canadensis*	Six live animals
	*C. elaphus*	Three live animals
	*Dama dama*	NCSM 14256*
	*Odocoileus hemionus*	USNM (NAN)
	*O. virginianus*	NCSM 298, 2678*; One live animal
Delphinidae	*Delphinus delphis*	USNM (NAN)
	*Tursiops truncatus*	NCSM 8217
Eschrichtiidae	*Eschrichtius robustus*	USNM (NAN)
Giraffidae	*Giraffa camelopardalis*	USNM 163312, 252547; Seven live animals
	*Okapia johnstoni*	One live animal
Monodontidae	*Delphinapterus leucas*	AKM (NAN)
	*Monodon monoceros*	AMNH M73314/16
Phocoenidae	*Phocoena phocoena*	USNM (NAN)
Physeteridae	*Physeter catodon*	NCSM 3281
Platanistidae	*Platanista gangetica*	AMNH (NAN)
Pontoporiidae	*Pontoporia blainvillei*	USNM (NAN)
Suidae	*Sus scrofa*	FSU (NAN); NCSM 16917
Tayassuidae	*Pecari tajacu*	AMNH 17352; USNM 14081, (NAN)
	*Tayassu pecari*	USNM 160652, 258578, 259091
Tragulidae	*Tragulus napu*	USNM 49605, 49871
	*T. javanicus*	USNM (NAN); YPM (NAN)
Ziphiidae	*Mesoplodon mirus*	NCSM 401
**Order Chiroptera**		
Molossidae	*Eumops perotis*	NCSM 8649*
	*Molossus nigricans*	USNM (NAN)
	*Tadarida brasiliensis*	NCSM 8283, 10392, 14971
Natalidae	*Natalus mexicana*	NCSM 8691
Phyllostomidae	*Artibeus jamaicensis*	AMNH 129101
	*Desmodus rotundus*	USNM (NAN)
	*Glossophaga soricina*	NCSM 8878, 8879
	*Leptonycteris sanborni*	NCSM 8693
Pteropodidae	*Pteropus edulis*	AMNH 245693
	*P. lylei*	AMNH 129100
	*P. samoensis*	USNM (NAN)
	*P. vampyrus*	NCSM 16329
Vespertilionidae	*Antrozous pallidus*	NCSM 5314*, 7704
	*Mormoops blainvillei*	NCSM 8035, 8058
	*Myotis velifer*	NCSM 8697, 8698, 8699; USNM (NAN)
**Order Eulipotyphla**		
Erinaceidae	*Atelerix albiventris*	NCSM 4588*, 45898*, 4590*, 5192*
Solenodontidae	*Solenodon paradoxus*	AMNH 269949
Soricidae	*Blarina brevicauda*	NCSM 324, 1888*, 1949, 13830, 14404, 14405, 14408
	*Notisorex crawfordi*	NCSM 9377*
	*Sorex cinereus*	NCSM 630, 17663
	*S. longirostris*	NCSM 13500*, 13501*, 13502*, 14589*, 14590*
Talpidae	*Condylura cristata*	NCSM 8509, 14632, 14633, 14636, 14656
	*Neurotrichus gibbsi*	NCSM 5353*, 6299, 7636*
	*Parascalops breweri*	NCSM 6152, 13303*, 14658
	*Scalopus aquaticus*	NCSM 004495, 8781, 17660
	*Scapanus townsendi*	NCSM 7635*, 7683*, 7794, 7998
**Order Hyracoidea**		
Procaviidae	*Dendrohyrax arboreus*	AMNH 55878*, 83246
	*D. dorsalis*	AMNH 52120, 53818*, 53806; USNM (NAN), 512790, 59852, 598583
	*Heterohyrax brucei*	AMNH 82100, 82102, 82104
	*Procavia capensis*	AMNH 35326, 35673; USNM 175011, 221377, 240928, 305093; YPM MAM 6838
**Order Lagomorpha**		
Leporidae	*Lepus arcticus*	AMNH 19169
	*L. callotis*	AMNH 1418
	*Oryctolagus cuniculus*	AMNH M144640
	*Sylvilagus floridanus*	NCSM 14102, 15652
Ochotonidae	*Ochotona pallasi*	AMNH 55981
	*O. princeps*	CM 9463, 16031, 20606; NCSM 8118, 8119
**Order Macroscelidea**		
Macroscelididae	*Elephantulus brachyrhynchus*	USNM 365027
	*E. intufi*	USNM 29153, 295149, 295158
	*E. rozeti*	USNM (NAN)
	*E. rufescens*	USNM 399312, 535125, 574953
	*Macroscelides proboscideus*	USNM 588428
	*Petrodromus tetradactylus*	USNM 241593, 365035; YPM MAM 10314
**Order Perissodactyla**		
Equidae	*Equus burchellii*	USNM 61743
	*E. caballus*	NCSM 433, 7675
Rhinocerotidae	*Dicerorhinus sumatrensis*	AMNH 54764
	*Rhinoceros sondaicus*	MCZ 5169 (photos), 5170 (photos); USNM 269392
	*R. unicornis*	MCZ 1730 (photos), 16893 (photos); USNM (NAN)
Tapiridae	*Tapirus bairdii*	USNM (NAN)
	*T. pinchaque*	USNM 11884
	*T. terrestris*	USNM 281726
**Order Pholidota**		
Manidae	*Manis longicaudata*	USNM (NAN)
**Order Primates**		
Atelidae	*Alouatta villosa*	USNM (NAN)
	*Ateles* sp.	USNM 47912, 49888
	*A. belzebuth*	AMNH 216*, 30637*, 98402*
	*A. fusciceps*	AMNH 32355*, 188139*
	*A. geoffroyi*	AMNH 17208*, 145158*; USNM (NAN), 102085
	*A. paniscus*	AMNH 17581*, 100076*
Callitrichidae	*Cebuella pygmaea*	USNM (NAN)
	*Saguinus oedipus*	USNM (NAN)
Cebidae	*Cebus capucinus*	USNM (NAN)
	*Cebus* sp.	NCSM 8363
Cercopithecidae	*Nasalis larvatus*	USNM (NAN)
	*Papio sphinx*	USNM (NAN)
	*Presbytis comate*	NCSM 16333, 16334
Cynocephalidae	*Cynocephalus volans*	USNM (NAN)
Daubentoniidae	*Daubentonia madagascariensis*	USNM (NAN)
Galagidae	*Galago senegalensis*	USNM (NAN)
Hominidae	*Gorilla gorilla*	USNM (NAN)
	*Homo sapiens*	FSU (NAN); NCSM 1214
	*Pan troglodytes*	USNM 48184, 176226, 220068, 220326, 220327, 236883, 236971, 256973, 395820
Hylobatidae	*Hylobates moloch*	USNM (NAN)
	*Symphilangus symphilangus*	USNM 49656
Lemuridae	*Eulemur mongoz*	USNM (NAN)
Lorisidae	*Arctocebus aureus*	USNM 598476*
	*A. calabarensis*	AMNH 212576, 212954; USNM 511930*
	*Loris lydekkerianus*	USNM 305067, 114692*, 256737*
	*Nycticebus bengalensis*	USNM 270994*, 39654
	*Perodicticus potto*	AMNH 15972; USNM 49547, 84227*, 184230*, 184229,*, 270530, 253619, 598550
Pitheciidae	*Cacajao calvus*	USNM (NAN)
Pongidae	*Pongo abelii*	USNM 49856
Tarsiidae	*Cephalopachus bancanus*	AMNH 2458
**Order Proboscidea**		
Elephantidae	*Loxodonta africana*	USNM 49489
**Order Rodentia**		
Abrocomidae	*Abrocoma cinerea*	USNM 583254
Anomaluridae	*Anomalurus beecrofti*	USNM 84546
	*A. pelii*	CM 69351
	*Idiurus zenkeri*	AMNH 56622
Aplodontiidae	*Aplodontia rufa*	NCSM 3770*, 4829*
Castoridae	*Castor canadensis*	NCSM 8518; USNM (NAN)
Caviidae	*Cavia porcellus*	USNM 35083
	*Dolichotis patagonum*	NCSM 8200*; USNM 175890, 258569
	*Galea spixii*	USNM 399272, 538313
	*Hydrochoerus hydrochaerus*	USNM 49456, 155412, 269946; USNM (NAN)
	*Kerodon rupestris*	USNM 399280, 543101
	*Microcavia australis*	USNM 54417, 132278
Chinchillidae	*Chinchilla chinchilla*	USNM 219408, 279438; one live animal
	*Lagidium peruanum*	USNM 194472, 194473
	*Lagostomus trichodactylus*	USNM 154146, 173042
Cricetidae	*Baiomys taylori*	NCSM 15106*, 15107*
	*Cleithrionomys gapperi*	NCSM 5165, 5836*, 5837*
	*Lemmus trimucronatus*	NCSM 2524*
	*Mesocricetus auratus*	NCSM 15808*
	*Microtus pinetorum*	NCSM 8515, 8901, 13350*, 13351*, 15586, 17665
	*Myodes gapperi*	NCSM 15573
	*M. rutilus*	NCSM 3252*
	*Neofiber alleni*	NCSM 1688*, 1689*, 3020*, 3021*
	*Neotoma floridana*	NCSM 2814*, 3723*
	*Ondatra zibethicus*	NCSM 374*, 4003*, 4008*, 6588*, 8265, 15104, 17664*
	*Oryzomys palustris*	NCSM (NAN), 499*, 500*, 501*, 17662
	*Peromyscus floridanus*	NCSM 2191*, 12027*
	*P. maniculatus*	NCSM 5621*, 5623*, 5625*, 15530
	*Reithrodontomys megalotis*	NCSM 5878*, 5879*
	*Sigmodon hispidus*	NCSM 12021*, 12023*, 12025*, 15635
	*Synaptomys cooperi*	NCSM 15585*, 17202*
Ctenomyidae	*Ctenomys* sp.	USNM 147922
Cuniculidae	*Cuniculus paca*	USNM 13057, 155610
Dasyproctidae	*Dasyprocta azarae*	AMNH 37457*, 134215; USNM 252297
	*D. fuliginosa*	AMNH 18841*, 35438
	*D. leporina*	AMNH 37151*, 80250, 265955
	*D. mexicana*	AMNH 172283*; USNM 49736
	*D. punctata*	AMNH 215102, 215099*; USNM 261397, 503777
	*Myoprocta acouchy*	AMNH 94073*, 70198
	*M. pratti*	AMNH 33654*
Dinomyidae	*Dinomys* sp.	USNM 300797, 395160
	*Dinomys branickii*	USNM 395453
Dipodidae	*Allactaga elater*	AMNH 212116
	*A. pumilio*	AMNH 85331*, 98133
	*A. sibirica*	AMNH 57227*, 58715*
	*Cardiocranius paradoxus*	AMNH 122*
	*Dipus sowerbyi*	AMNH 176265*
	*Eozapus setchuanus*	AMNH 84264*
	*Jaculus jaculus*	AMNH 70096
	*J. orientalis*	AMNH 525*
	*Napeozapus insignis*	AMNH 67768, 121830*; NCSM 15589
	*Salpingotus thomasi*	AMNH 249428*
	*Zapus hudsonius*	AMNH 206850; NCSM 2559
	*Z. princeps*	AMNH 238252*
	*Z. trinotatus*	AMNH 1244*, 38311*
Echimyidae	*Cercomys cuniculus*	USNM 543479
	*Hoplomys gymnurus*	USNM 578393
	*Proechimys canicolli*	USNM 280054
	*Thrichomys apereoides*	NCSM 12964*; USNM 293173
Erethizontidae	*Coendou* sp.	USNM 267592, 297843
	*Erethizon dorsatum*	NCSM 4748*, 6213, 7825, 13040*, 16262*; USNM 88617, 568394, 568395
Geomyidae	*Geomys bursarius*	NCSM 15078*, 15080*
	*G. pinetus*	NCSM 1787*, 2143*
	*Thomomys bottae*	NCSM 5905*
	*Th. talpoides*	NCSM 5892*, 5897*
Heteromyidae	*Chaetodipus californicus*	NCSM 882*
	*Ch. baileyi*	NCSM 2993*
	*Dipodomys ordii*	NCSM 5257*, 5868*
	*Peromyscus pencillatus*	NCSM 9902*, 9905*
Hystricidae	*Atherurus africanus*	USNM 539828, 538109
	*A. macrourus*	USNM 49498, 49602
	*Hystrix brachyura*	USNM 197641, 153974, 49465
	*H. crassispinis*	USNM 153974, 197640, 396591
	*H. cristata*	USNM 142163, 538408
	*H. indica*	USNM 60073, 570871
	*H. javanica*	USNM 155287
	*H. sumatrae*	USNM 49870, 49932
	*Trichys fasciculata*	USNM 347835
Muridae	*Acomys dimidiatus*	NCSM 15804* (two skins with same number)
	*Gerbillurus paeba*	USNM 295264
	*Gerbillus* sp.	NCSM 15858*, 15859*
	*Meriones unguiculatus*	USNM 290460
	*Mus musculus*	NCSM 5723*, 8774, 8775, 15647, 15864*
	*Psammomys obesus*	USNM 308354
	*Rattus norvegicus*	NCSM (NAN), 201*, 202*, 203*, 1207; USNM 308359, 564244
	*Tatera indica*	USNM 329220
Myocastoridae	*Myocastor coypus*	NCSM 299*, 1109*
Octodontidae	*Octodon degus*	USNM 397332
Pedetidae	*Pedetes capensis*	USNM 49647, 221381, 384097
Sciuridae	*Ammospermophilus harrisi*	NCSM 2250*, 9834*
	*Cynomys gunnisoni*	NCSM 6406*, 6412*, 15387
	*Eutamias cinereicollis*	NCSM 5925*
	*Glaucomys volans*	NCSM 91*, 730*, 9860*, 7315*, 14985, 16805, 16807
	*Marmota monax*	NCSM 7218*, 7517*, 7771*, 9680; USNM (NAN)
	*Sciurus carolinensis*	NCSM 5247, 12909, 14990*, 16873*, 16874, 16875*, 17685*
	*S. niger*	NCSM 8491, 17306*, 17307*, 17664*, NAN*
	*Spermophilus beecheyi*	NCSM 9811*, 9812*
	*S. lateralis*	NCSM 5922*, 5923*, 5924*, 9814*
	*S. richardsoni*	NCSM 6411*
	*Tamias striatus*	NCSM 8096, 15491, 16382*, 16385*
	*Tamiasciurus hudsonicus*	NCSM 8383, 15492
Thryonomyidae	*Thryonomys swinderianus*	AMNH 241385, 341383*
	*Th. gregorianus*	USNM 300796, 318094
**Order Scandentia**		
Tupaiidae	*Tupaia* sp.	AMNH 70299
	*T. glis*	NCSM 9386*, 9387*; USNM (NAN)
**Order Sirenia**		
Dugongidae	*Dugong dugon*	AMNH (NAN); USNM (NAN)
Trichechidae	*Trichechus manatus*	NCSM 4566, 4569, 4571, 4572; USNM (NAN), 14334 (photos), 217259 (photos)
	*T. inunguis*	USNM 20916 (photos)
**Order Tubulidentata**		
Orycertopodidae	*Orycteropus afer*	USNM (NAN)
**Order Xenarthra**		
Bradypodidae	*Bradypus tridactylus*	USNM 256676
	*B. variegatus*	USNM 49590
Cyclopedidae	*Cyclopes didactylus*	NCSM 16252*; USNM 283876, 583607
Dasypodidae	*Chaetophractus villosus*	AMNH 240; USNM 302063; USNM (NAN)
	*Chlamyphorus truncatus*	AMNH (NAN)
	*Dasypus novemcinctus*	NCSM 7353*, 7354*, 9059*, 9060*, 16454
	*Priodontes maximus*	AMNH (NAN); USNM 261024
	*Tolypeutes matacus*	USNM 291935
Megalonychidae	*Choloepus didactylus*	USNM 256769
	*Ch. hoffmanni*	USNM 012859; USNM (NAN)
Myrmecophagidae	*Myrmecophaga tridactyla*	AMNH 1873; USNM (NAN)
	*Tamandua tetradactyla*	AMNH 238, M385

Rodentia is the largest mammalian order, with over 1,700 species, of which over 70% are in the superfamily Muroidea (mice and kin) and the family Sciuridae (squirrels and kin) (Adkins, Walton & Honeycutt, 2003; Steppan, Storz & Hoffmann, 2004). Therefore, we were able to examine only a fraction of the diversity within those two taxa. Nevertheless, we achieved sufficient coverage of them to be of use here. Our overall rodent sample includes representatives of 23 (82%) of the 28 extant rodent families listed by Nowak (1999).

### Identification of vestigial structures

Three categories of skeletal structures were examined and considered candidates for identification as vestigial structures: (1) individual bones, (2) parts of individual bones (e.g., the shaft of the ulna), and (3) multiple-bone structures (e.g., the pelvic girdle, a limb, or a digit). Previous studies have identified vestigiality in all three anatomical categories (Tague, 1997; Kearney, 2002; Bejder & Hall, 2002; Maxwell & Larsson, 2007; Senter, 2010; Bensimon-Brito et al., 2011; Moch & Senter, 2011).

We began by identifying examples of postcranial skeletal structures that were greatly reduced in comparison to their homologs in related taxa. To determine whether such structures could be considered vestigial we used the phylogenetic bracketing approach from a previous study (Moch & Senter, 2011). According to this approach, a structure is considered vestigial if it satisfies the three criteria listed below, in comparison to its homolog in three successive sister taxa (Table 2). A phylogenetic bracketing approach only requires confirmation of a character state in two successive sister taxa (Witmer, 1995), but we included a third so as to increase the reliability of the inference.

**Table 2:** Outgroup lists. Vestigial skeletal structures in mammalian taxa, and successive outgroups with unreduced homologs of those structures, demonstrating that such structures are vestigial. The symbol † indicates an extinct taxon. To determine the succession of outgroups, we used the phylogenies in the references given in the caption to Fig. 1, with additional information from Thewissen et al. (2007).

Taxon exhibiting vestigial structure	Vestigial structure	Outgroup 1	Outgroup 2	Outgroup 3
**Clade Metatheria**				
**Order Dasyuromorphia**				
*Antechinomys*	Toe I	*Dasyurus*	Peramelidae	Caenolestidae
*Dasyuroides*	Toe I	*Dasyurus*	Peramelidae	Caenolestidae
*Sarcophilus*	Toe I	*Dasyurus*	Peramelidae	Caenolestidae
*Thylacinus*	Metatarsal I	*Dasyurus*	Peramelidae	Caenolestidae
**Order Peramelemorphia**				
*Chaeropus ecaudatus*	Finger IV	Peramelidae	Dasyuromorphia	Caenolestidae
	Toe II	Peramelidae	Dasyuromorphia	Caenolestidae
	Toe III	Peramelidae	Dasyuromorphia	Caenolestidae
	Toe V	Peramelidae	Dasyuromorphia	Caenolestidae
**Clade Eutheria**				
**Order Carnivora**				
Canidae	Clavicle	Chiroptera	Primates	Pilosa
	Toe I	Ursidae	Chiroptera	Primates
Felidae	Clavicle	Chiroptera	Primates	Cingulata
	Metatarsal I	Ursidae	Chiroptera	Primates
*Crocuta* + *Hyaena*	Finger I	*Proteles*	Felidae	Ursidae
Hyaenidae	Toe I	Ursidae	Chiroptera	Primates
**Clade Cetartiodactyla**				
Antilocapridae	Metatarsal V	Tragulidae	Hippopotamidae	Suidae
Camelidae	Fibula	Suidae	Rhinocerotidae	Ursidae
Cervidae	Metacarpal II	Tragulidae	Hippopotamidae	Suidae
	Metacarpal V	Tragulidae	Hippopotamidae	Suidae
	Metatarsal II	Tragulidae	Hippopotamidae	Suidae
	Metatarsal V	Tragulidae	Hippopotamidae	Suidae
Cetacea (crown clade)	Pelvic girdle	*Rodhocetus* ^†^	*Ambulocetus* ^†^	*Indohyus* ^†^
Delphininae	Finger IV	Monodontidae	Ziphiidae	*Physeter*
	Finger V	Ziphiidae	Mysticeti	Basilosauridae^†^
*Giraffa camelopardalis*				
	Metatarsal II	Tragulidae	Hippopotamidae	Suidae
Globicephalinae	Finger IV	Monodontidae	Ziphiidae	*Physeter*
	Finger V	Ziphiidae	Mysticeti	Basilosauridae^†^
*Inia*	Finger V	Ziphiidae	Mysticeti	Basilosauridae^†^
Mysticeti	Sternum	Odontoceti	Basilosauridae^†^	*Ambulocetus* ^†^
	Hindlimb	*Rodhocetus* ^†^	*Ambulocetus* ^†^	*Indohyus* ^†^
*Okapia johnstoni*	Metatarsal V	Tragulidae	Hippopotamidae	Suidae
Pandelphina +				
Ziphiidae	Finger I	Mysticeti	Basilosauridae^†^	*Rodhocetus* ^†^
Pecora	Fibula	Tragulidae	Hippopotamidae	Suidae
*Phocoena*	Finger V	Ziphiidae	Mysticeti	Basilosauridae^†^
Tayassuidae	Metatarsal V	Suidae	Hippopotamidae	Ursidae
**Order Chiroptera**	Ulnar shaft	Carnivora	Primates	Xenarthra
**Order Hyracoidea**	Finger I			
	Metatarsal V	Tubulidentata	Primates	Didelphidae
**Order Lagomorpha**	Clavicle	Castoridae	Primates	Chiroptera
Leporidae	Metatarsal I	Castoridae	Primates	Chiroptera
**Order Macroscelidea**				
*Elephantulus* +				
*Macroscelides* +				
*Petrodromus*	Ulnar shaft	*Rhynchocyon*	Tubulidentata	Proboscidea
**Order Perissodactyla**				
*Equus*	Ulnar shaft	*Orohippus* ^†^	*Hyracotherium* ^†^	Rhinocerotidae
	Metacarpal II	*Merychippus* ^†^	*Orohippus* ^†^	*Hyracotherium* ^†^
	Metacarpal IV	*Merychippus* ^†^	*Orohippus* ^†^	*Hyracotherium* ^†^
	Fibula	*Orohippus* ^†^	*Hyracotherium* ^†^	Rhinocerotidae
	Metatarsal II	*Merychippus* ^†^	*Orohippus* ^†^	*Hyracotherium* ^†^
	Metatarsal IV	*Merychippus* ^†^	*Orohippus* ^†^	*Hyracotherium* ^†^
Rhinocerotidae	Metatarsal I	*Phenacodus* ^†^	Ursidae	Pholidota
+ Tapiridae				
**Order Primates**				
Arctocebus +				
*Perodicticus*	Finger II	*Loris*	Galagidae	Lemuridae
*Ateles*	Metacarpal I	*Alouatta*	Cebidae	Pitheciidae
*Brachyteles*	Metacarpal I	*Alouatta*	Cebidae	Pitheciidae
*Colobus*	Metacarpal I	Cercopithecidae	Hominoidea	Tarsiidae
Hominoidea	Tail	Cercopithecoidea	Platyrrhini	Tarsiidae
**Order Rodentia**				
Caviidae	Metacarpal I	*Abrocoma*	*Atherurus*	*Bathyergus*
	Metatarsal I	*Ctenomys*	*Hystrix*	*Aplodontia*
	Metatarsal V	*Ctenomys*	*Hystrix*	*Thryonomys*
Chinchillidae	Metatarsal I	*Ctenomys*	*Hystrix*	*Aplodontia*
*Coendou* +				
*Erethizon*	Finger I	*Abrocoma*	*Atherurus*	*Bathyergus*
Dasyproctidae	Metatarsal I	*Ctenomys*	*Hystrix*	*Aplodontia*
*Dipodomys*	Metatarsal I	*Rattus*	*Castor*	Primates
*Hystrix crassispinis*	Finger I	*Hystrix indica*	*Atherurus*	*Abrocoma*
*Jaculus*	Metatarsal I	*Zapus*	*Rattus*	*Castor*
*Lagidium* +				
*Lagostomus*	Metacarpal I	*Abrocoma*	*Atherurus*	*Bathyergus*
*Lagostomus*	Metatarsal V	*Ctenomys*	*Hystrix*	*Thryonomys*
Pedetidae	Metatarsal I	*Idiurus*	*Castor*	*Rattus*
Thryonomyidae	Toe I	*Hystrix*	*Aplodontia*	*Castor*
**Order Sirenia**	Finger I	Tenrecidae	Primates	Didelphidae
	Pelvic girdle	Proboscidea	Hyracoidea	Tubulidentata
**Order Xenarthra**				
*Bradypus*	Metacarpal I	Primates	Tenrecidae	Didelphidae
	Metacarpal V	*Chlamyphorus*	Primates	Tenrecidae
	Metatarsal I	*Chlamyphorus*	Primates	Tenrecidae
	Metatarsal V	*Chlamyphorus*	Primates	Tenrecidae
*Choloepus*	Metacarpal I	Primates	Tenrecidae	Didelphidae
	Metacarpal IV	*Bradypus*	*Chlamyphorus*	Primates
	Metatarsal I	*Chlamyphorus*	Primates	Tenrecidae
	Metatarsal V	*Chlamyphorus*	Primates	Tenrecidae
*Cyclopes*	Metacarpal I	Myrmecophagidae	*Chlamyphorus*	Primates
*Dasypus*	Finger V	*Chaetophractus*	Primates	Tenrecidae
*Myrmecophaga*	Manual phalanx V-2	*Chaetophractus*	Primates	Tenrecidae
*Priodontes maximus*	Finger V	Primates	Tenrecidae	Didelphidae
*Tamandua*	Finger V	*Chaetophractus*	Primates	Tenrecidae
*Tolypeutes matacus*	Metacarpal I	Primates	Tenrecidae	Didelphidae
	Metacarpal V	Primates	Tenrecidae	Didelphidae

The first criterion for vestigiality is that in comparison to its state in the sister groups the structure exhibits extreme reduction. For this study, we considered this criterion met if the structure was reduced to one-third its size relative to adjacent skeletal structures, in comparison with its state in the sister groups. This fraction is arbitrary and is not necessarily applicable to other studies; we used it here simply to have a consistent standard for extreme reduction. We used fossil taxa as sister groups for comparison in the Cetacea and Perissodactyla. We used extant taxa as sister groups in all other cases (Table 2).

The second criterion is that the structure has lost the specialized morphology that it exhibits in the sister groups. For example, a finger meets this criterion if its distal phalanx is shaped like an ovoid pebble in the taxon in question but has the form of an ungual (a claw-bearing phalanx) in the sister groups.

The third criterion is that the structure has lost a salient ancestral function. Although it may not be completely functionless, biologists consider it vestigial only if it has lost a major function (e.g., Darwin, 1859; Wilson, 1982; Prothero, 2007; Hall & Hallgrimsson, 2008; Bergstrom & Dugatkin, 2012). A limb satisfies this criterion, for example, if it is too reduced to serve as an organ of propulsion, whereas it is an organ of propulsion in the sister groups and therefore arguably in their common ancestor. Likewise, a pelvic girdle satisfies this criterion if it is too reduced to anchor a full limb and the muscles that operate it for propulsion, whereas in the sister groups it anchors a full limb and propulsive muscles. A pelvic girdle further satisfies this criterion if it is not connected to the vertebral column, because such a connection facilitates propulsion with the hindlimb by ensuring that each step propels the entire vertebral column (Kardong, 2008). A digit satisfies this criterion if it is too reduced for the functions of prehension or bodily support.

It is important to confirm that apparent vestigiality is characteristic of a species and not simply due to aberrance in a single specimen. Therefore, when we found reduced structures in taxa for which, to our knowledge, vestigial structures had not been previously documented, we examined more than one specimen per species when possible (Table 1). This also revealed individual variation, which is important because vestigial structures are often highly variable (Darwin, 1859; Omura, 1980; Conrad, 1982; Tague, 1997). For suprageneric taxa in which adequate previous descriptions of vestigial structures existed, we examined fewer specimens so as to spend a greater fraction of available time on previously undocumented or undescribed vestigial structures (Table 1). For the same reason, we also examined fewer specimens per species of suprageneric taxa lacking vestigial structures.

For this study we examined tail skeletons, clavicles, forelimb bones, pelvic girdles, and hindlimb bones. It is possible that vestigial skeletal structures are identifiable in other parts of the mammalian skeleton, e.g., the skull, parts of vertebrae, parts of the scapula, thyroid bones, cardiac bones, and the baculum. Such were not included in this study but may prove fruitful avenues for future research.

### Tracing phylogenetic patterns

We used the cladogram in Fig. 1 (see caption for information sources) to trace phylogenetic patterns in vestigiality and loss of skeletal structures. Onto this cladogram we mapped the phylogenetic distribution of vestigiality and loss in postcranial skeletal elements, as shown in the figure. We then used this mapping, plus information from the fossil record as needed, to determine the phylogenetic points at which vestigiality or loss occurred for given skeletal elements. In this determination we used two assumptions. The first assumption is that if all members of a clade share a character state (e.g., vestigiality or loss of a skeletal structure), then that state arose in the clade’s common ancestor. For example, if the second toe is vestigial in all members of a clade, then it was vestigial in the clade’s common ancestor. The second assumption is that vestigialization and loss are not reversible. Therefore, if the second toe is lost in two clades but is present in a clade that is phylogenetically bracketed by the two clades, then the two clades lost the second toe independently instead of having inherited that loss from a common ancestor.

Missing data and parallel evolution cause challenges when character states (traits) are mapped onto phylogenies, making it difficult to distinguish convergences (in which two or more lineages gain the same character state) from reversals (in which members of a taxon revert to a previous character state). Software for phylogenetic analysis often resolves the problem by presenting two alternate solutions: one that interprets the phylogenetic pattern according to the principle of accelerated transformation (in which reversals are considered more likely than convergences) and one that interprets the phylogenetic pattern according to the principle of delayed transformation (in which convergences are considered more likely than reversals) (Maddison & Maddison, 1992). The two principles yield identical results when patterns of changes in character states are unambiguous. Our data set is sufficiently simple that changes in character states are unambiguous in most cases, obviating the need for software. However, the reader should note that because we assumed non-reversibility of loss, we used the principle of delayed transformation in the few cases that did exhibit ambiguity. For example, in the case of vestigialization of the clavicle, we used the principle of delayed transformation because of a problem with missing data, i.e., tiny clavicles that are present in the live animal are often missing or overlooked in disarticulated museum specimens (see the Clavicle section of the Results). For other bones, there were no problems with missing data. Similarly, disagreement in the literature regarding rodent phylogeny engendered ambiguity in the interpretation of the evolution of vestigialization of the hallux (the first toe) of rodents, and we used the principle of delayed transformation to interpret the results according to multiple possible phylogenies (see the Foot and toe section of the Results). For other mammalian taxa, there were no problems with phylogenetic disagreement.

Our employment of the assumption that losses are irreversible deserves further comment. There are exceptions to the general rule that vestigiality and loss are irreversible. In iguanodontian dinosaurs the ancestrally-vestigial fifth finger became elongated and useful for grasping (Senter, 2010), and atavistic limbs in aberrant cetacean and sirenian individuals show that loss is not completely irreversible (Abel, 1908; Andrews, 1929; Ogawa & Kamiya, 1957; Ohsumi, 1965). Lost body parts can be regained in the case of meristic traits in which the number of repeated parts is determined late in ontogeny (Galis, Arntzen & Lande, 2010). However, regaining of lost structures is rare (Goldberg & Igić, 2008; Galis, Arntzen & Lande, 2010), and there is no evidence that it has occurred within the Mammalia except in such atavistic cases.

## Results

### Mammalian taxa without vestigial skeletal structures

There are a few major mammalian clades among whose extant representatives we did not find vestigial structures in the postcranial skeleton. These are the Monotremata (platypus and echidna); marsupial orders other than Dasyuromorphia and Peramelemorphia; and the eutherian orders Tubulidentata (aardvark), Proboscidea (elephants), Pholidota (pangolins), Eulipotyphla (shrews, moles, hedgehogs, and kin), and Scandentia (tree shrews).

### Sternum

**Figure 2:**
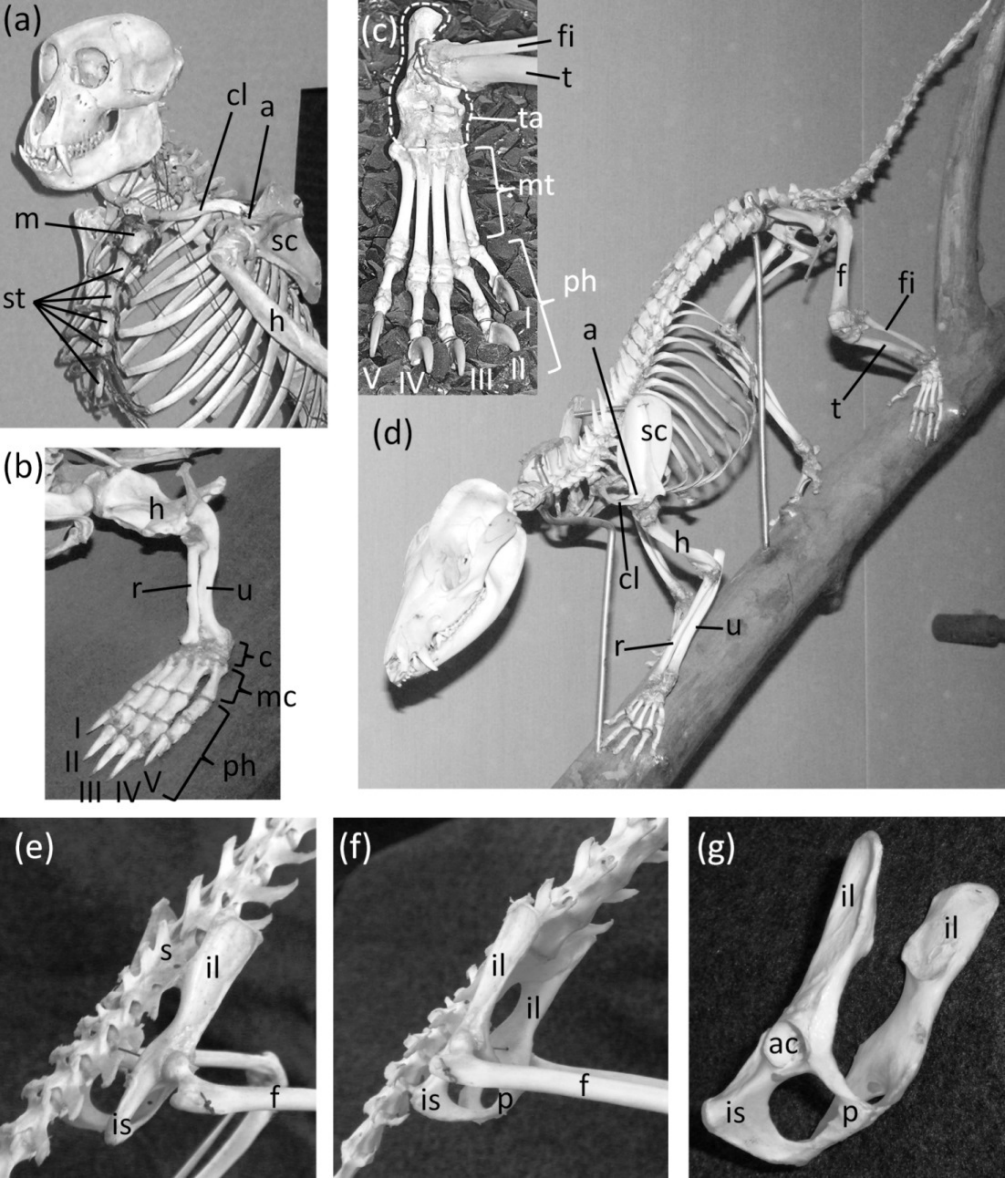
Mammal skeletons with structures in a non-vestigial state. (A) *Nasalis larvatus* (proboscis monkey), showing full expression of the clavicle and sternum (B) *Ornithorhynchus anatinus* (platypus), showing full expression of all five digits of the hand, with two phalanges in the thumb and three in each other finger (C) *Procyon lotor* (raccoon), showing full expression of all five digits of the foot, with two phalanges in the first toe and three in each other toe (D) *Didelphis virginiana* (Virginia opossum), showing full expression of the shafts of the ulna and fibula (E) *Saimiri* sp. (squirrel monkey) in right dorsolateral view, showing full expression of the pelvic girdle (F) *Saimiri* sp. (squirrel monkey) in right ventrolateral view, showing full expression of the pelvic girdle and its attachment to the sacrum (G) Pelvic girdle of *Felis catus* (domestic cat) in right ventrolateral view, showing the parts of a fully-expressed pelvic girdle.

Ancestrally, the mammalian sternum consists of several segments called sternebrae, the first of which is called the manubrium (Fig. 2). The ribs articulate with the sternum via cartilaginous extensions called costal cartilages.

**Figure 3:**
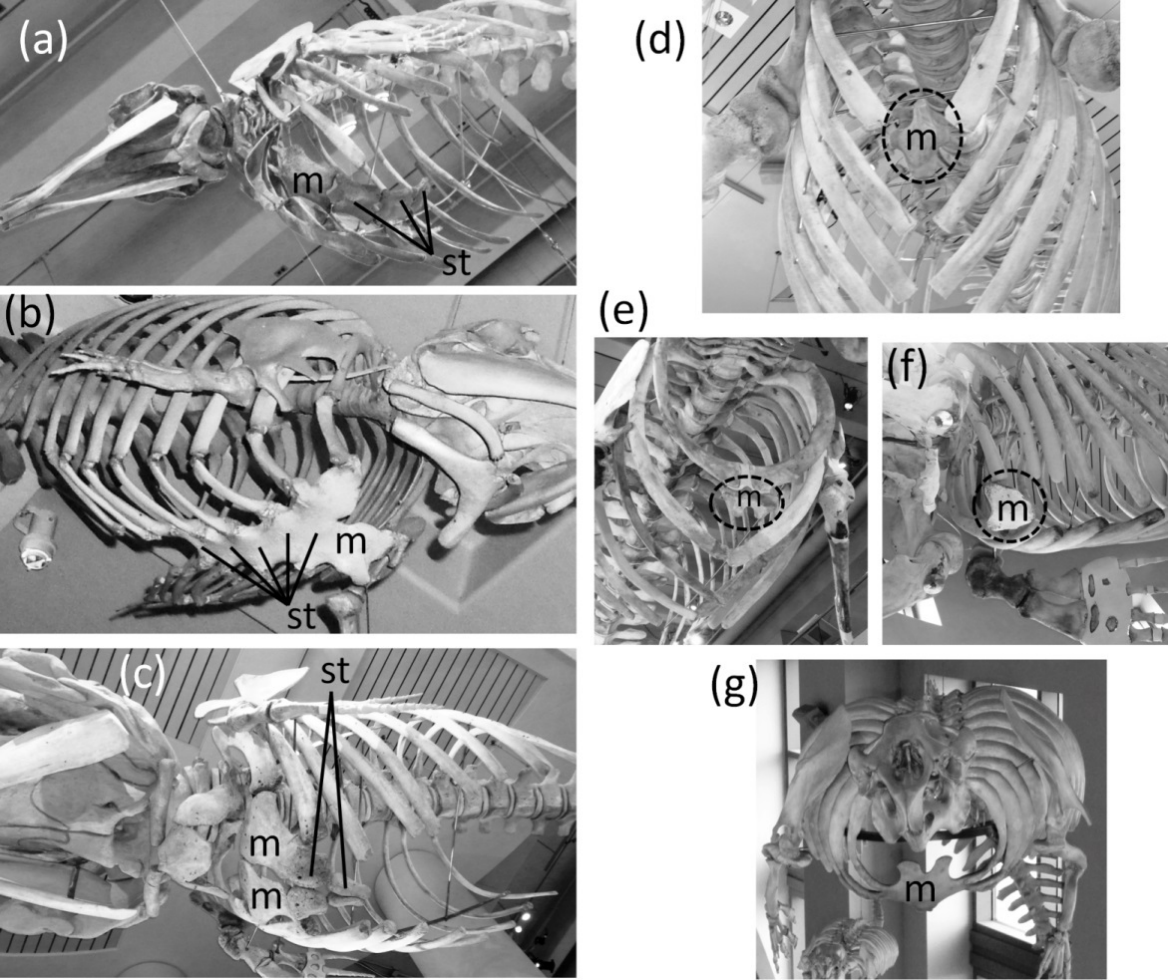
Sterna of whales, with vestigial parts circled with broken line. (A) Non-vestigial sternum of *Mesoplodon mirus* (True’s beaked whale) (B) Non-vestigial sternum of *Monodon monoceros* (narwhal), with segments fused into one, which is common in toothed whales (C) Sternum of *Physeter catodon* (sperm whale) with reduced post-manubrial section (D) Vestigial sternum of *Balaenoptera musculus* (blue whale) (E) Vestigial sternum of *Megaptera novaeangliae* (humpback whale) (F) Vestigial sternum of *Eubalaena glacialis* (North Atlantic right whale); (G) Non-vestigial sternum of *Trichechus manatus* (West Indian manatee).

In most Odontoceti (toothed whales) most of the ribs maintain their connection with the sternum, and the sternum is unreduced (Fig. 3). An exception is *Physeter catodon* (sperm whale), in which the post-manubrial sternum has lost all but two sternebrae. They are reduced in relative size, but not enough to satisfy the first criterion for vestigiality (Fig. 3C).

In the Mysticeti (baleen whales) most of the ribs have lost the costal cartilages and are therefore no longer connected to the sternum. This enables the ribcage to collapse more than is possible in other mammals, so that a greater amount of air can be expelled from the lungs for deep diving. The sternum—which would prevent such ribcage collapse if it were fully expressed—is vestigial. Only the manubrium is retained (Figs. 3D–3F) (Howell, 1930).

In the Sirenia (manatees and dugongs) the number of segments in the sternum is also reduced. Reduced rib mobility in sirenians prevents ribcage collapse (Howell, 1930), so there is less selection pressure to maintain a large sternum. The sternum retains the manubrium and a second ossification that appears to be homologous to the rest of the sternum but is reduced in size and undivided into sternebrae (Howell, 1930). However, the size of the sirenian sternum is not reduced enough to satisfy the first criterion for vestigiality (Fig. 3G), and because it maintains its cartilaginous connection to several ribs it also fails to satisfy the third criterion.

### Tail

Mammalian tails vary widely in length. Even short tails with a small number of vertebrae, such as those of many ungulates, perform important functions such as fly swatting and social signaling and therefore fail to satisfy the third criterion for vestigiality. It is therefore difficult to find examples of unambiguously vestigial tails in mammals.

**Figure 4:**
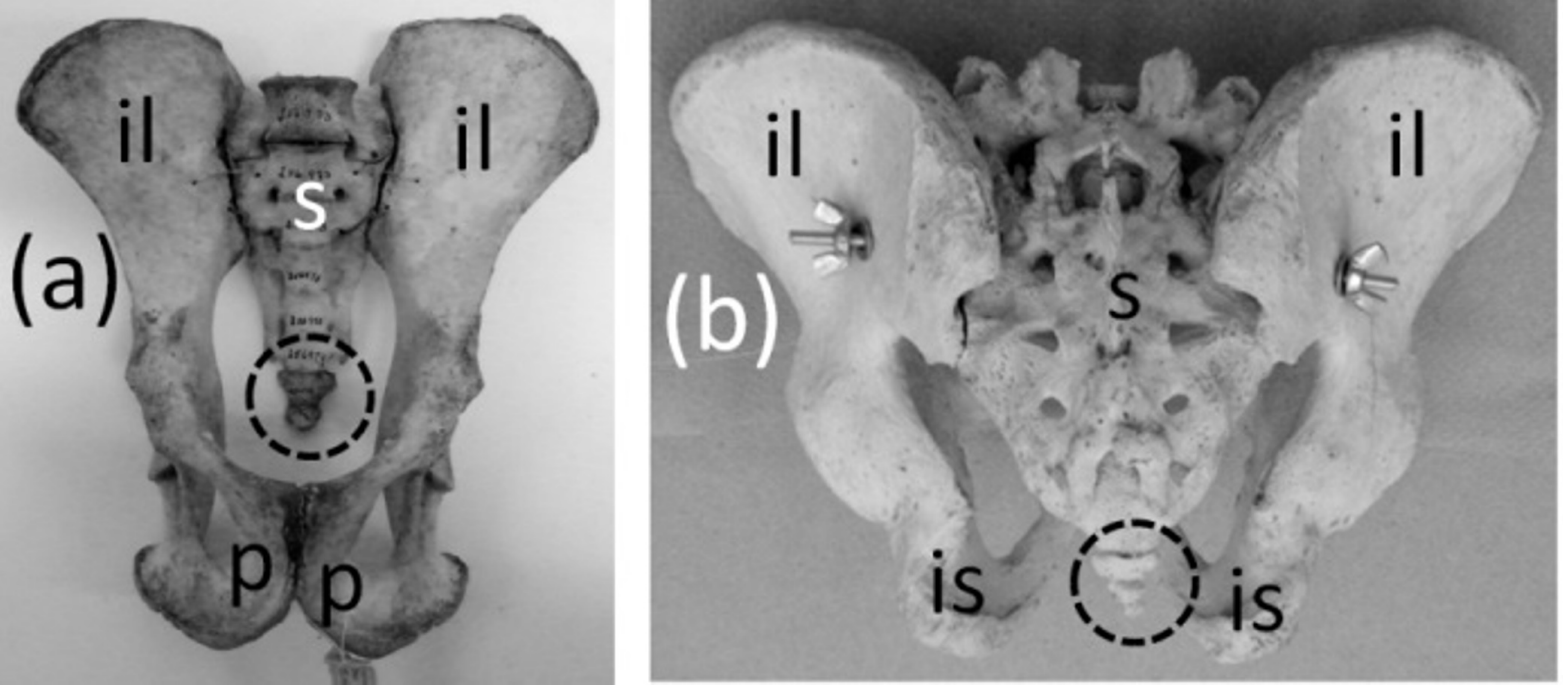
Pelves of primates, with coccyx (vestigial tail) circled with broken line. (A) *Pan troglodytes* (chimpanzee), ventral view (B) *Homo sapiens* (human), dorsal view.

The coccyx of apes and humans, a fused series of three to six vertebrae (Fig. 4), satisfies all three criteria for vestigiality. It does not protrude from the body’s surface and therefore cannot be used for social signaling, fly swatting, etc.

### Clavicle

Ancestrally, the mammalian clavicle is a robust bone that articulates with the sternum and the acromion process of the scapula, bracing the forelimb against the axial skeleton (Fig. 2A). Most mammals retain this condition, but some have lost the clavicle altogether. This loss facilitates forward motion of the scapula, which increases stride length during running (Ewer, 1973; Hildebrand & Goslow, 2001). The clavicle is lost in the orders Perissodactyla (odd-toed hoofed mammals), Cetartiodactyla (even-toed hoofed mammals and whales), Sirenia (manatees and dugongs), Hyracoidea (hyraxes), and Proboscidea (elephants); most members of Carnivora (carnivores); and some rodents (Flower, 1870).

**Figure 5:**
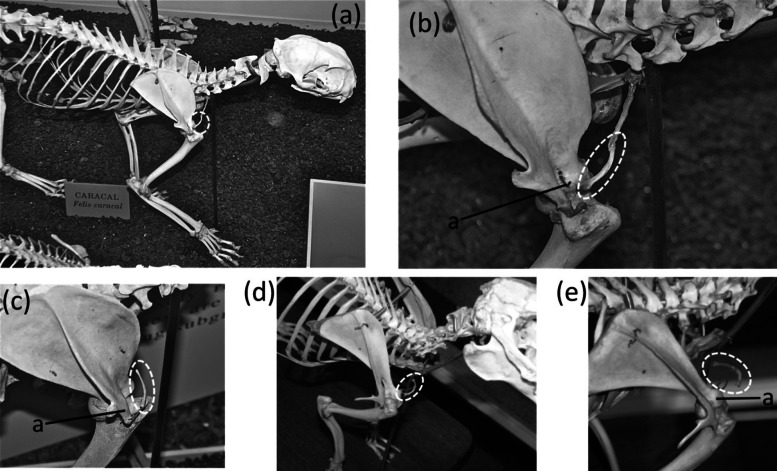
Vestigial clavicles, circled with broken line. (A) *Caracal caracal* (caracal). (B) *C. caracal*, close-up (C) *Acinonyx jubatus* (cheetah) (D) *Oryctolagus cuniculus* (domestic rabbit) (E) *O. cuniculus*, close-up.

A vestigial clavicle is retained in two carnivoran families: Canidae (the dog family) and Felidae (the cat family). In both, only a short sliver ossifies (Figs. 5A–5C) within a ligament that connects the sternum to the acromion and represents the degenerate remainder of the ancestral clavicle, and in the Canidae the clavicle is often absent (Ewer, 1973). A similar situation is present in rabbits (Figs. 5D–5E) (Flower, 1870).

A vestigial clavicle is present in some rodents, such as the guinea pig (*Cavia porcellus*), in which it is embedded in muscle, has no connection with the sternum, and has a loose attachment of fibrous tissue to the capsule of the shoulder joint (Cooper & Schiller, 1975). We did not confirm the presence of a vestigial clavicle in other rodents, because a vestigial clavicle is difficult to identify in osteological specimens. Due to its tiny size, a vestigial clavicle is easily missed in a box of disarticulated bones, and it is often missing on articulated skeletons because it does not articulate with other bones. Radiography of rodents, to determine the taxonomic distribution of vestigial clavicles, was prevented by logistical constraints during the course of this study but would make an interesting study for future researchers.

The phylogenetic distribution of character states (Fig. 1) indicates that the clavicle was independently lost at least four times in the Mammalia: once in the common ancestor of the Hyracoidea, Sirenia, and Proboscidea; once in the Cetartiodactyla; once in the Hyaenidae; and once in the common ancestor of the Ursidae, Mustelidae, and Procyonidae. The clavicle became vestigial independently in at least three groups: Lagomorpha, Felidae, and Canidae.

### Forearm

**Figure 6:**
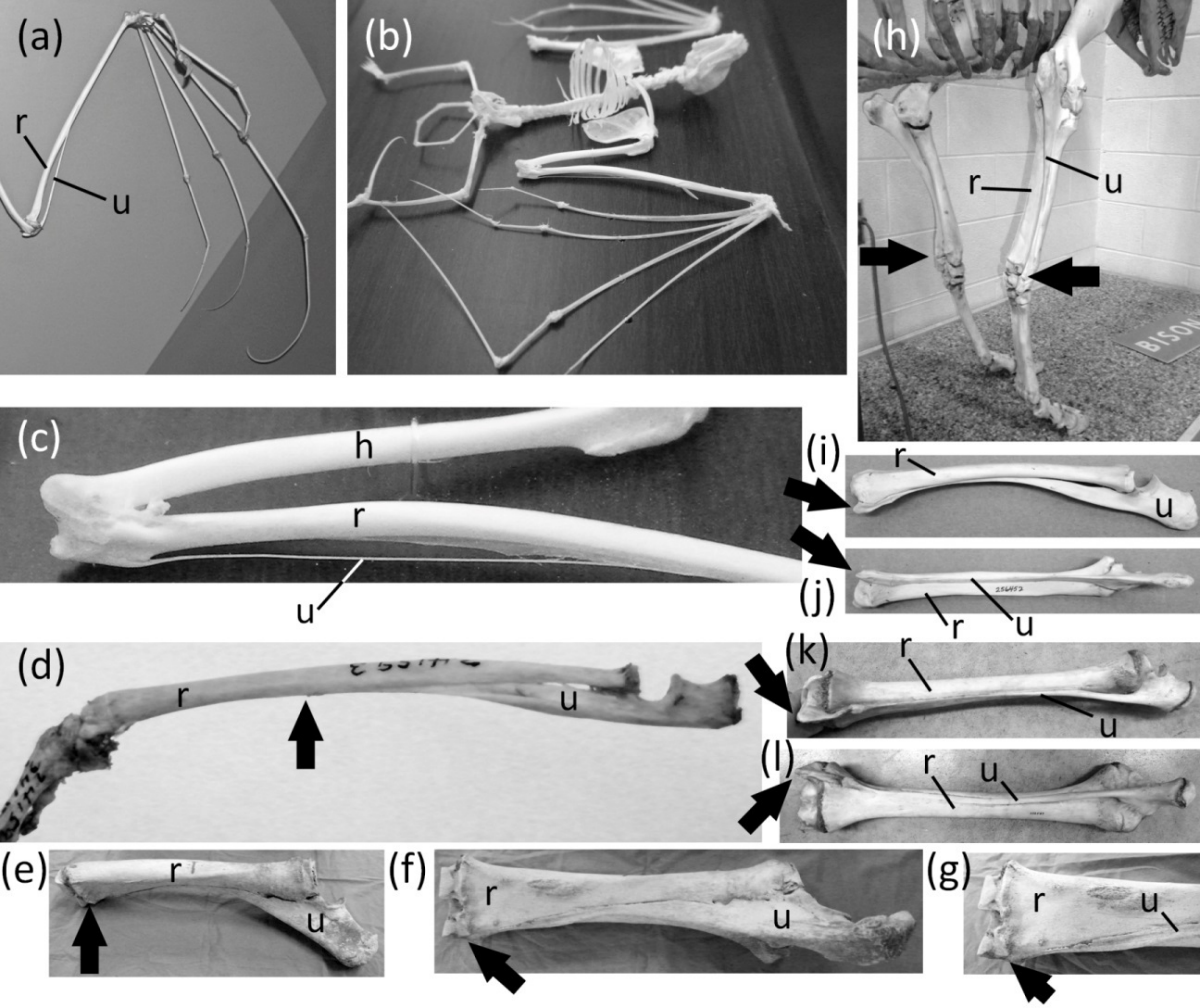
Vestigial ulnar shafts in bats, an elephant shrew, and a horse, and nearly-vestigial ulnae in artiodactyls, with distal tip of ulna indicated by arrow. (A) *Pteropus samoensis* (Samoan fruit bat) (B) *Pipistrellus abramus* (Japanese pipistrelle) (C) close-up of ulna of *P. abramus* (D) *Petrodromus tetradactylus* (four-toed elephant shrew) (E) right ulna of *Equus caballus* (domestic horse) in medial view (F) Same specimen as in *e*, in posterior view (G) Close-up of distal end of ulna in *f* (H) *Bison bison* (American bison) (I) *Antilocapra americana* (pronghorn), left forearm in lateral view (J) Same specimen as in *i*, extensor (posterior) view (K) *Giraffa camelopardalis* (giraffe), left forearm in lateroposterior view (L) Same specimen as in *k*, extensor (posterior) view.

Ancestrally, the mammalian forearm contains two bones, the radius and ulna. Each articulates with the humerus proximally and the carpus distally and functions as a strut between the carpus and humerus (Figs. 2B and 2D). The joint between the humerus and ulna is the hinge of the elbow; the proximal end of the ulna is therefore never lost in mammals. The shaft of the ulna, however, is reduced to a vestigial state in some mammals. In such cases the radius is the only strut between the carpus and humerus. Such is the case in the Chiroptera (bats); most Macroscelidea (elephant shrews); and *Equus* (horses). In bats the ulnar shaft is reduced to a threadlike sliver (Figs. 6A–6C). Among elephant shrews, the ulna is unreduced in the genus *Rhynchocyon*, but its shaft is vestigial in other genera, tapering to a point about halfway down the length of the radius (Evans, 1942) (Fig. 6D). In *Equus* the shaft of the ulna tapers to a point and does not reach the carpus (Nickel et al., 1986) (Figs. 6E–6G).

The ulnar shaft is reduced in the Camelidae (camels and kin) and ruminants. This reduction is extreme in the Camelidae and the ruminant families Giraffidae (giraffe and okapi), Cervidae (deer), Antilocapridae (pronghorn), and Bovidae (cattle, sheep, goats, and antelope). In these families the ulnar shaft is present for its full length but is reduced in transverse diameter to one-fourth or less the transverse diameter of the radial shaft, and the shafts of the two bones are co-ossified (Figs. 6I–6L). Because the ulnar shaft is present for its full length, it retains its ancestral function as a strut between the carpus and humerus and therefore does not satisfy the third criterion for vestigiality.

The phylogenetic distribution of character states (Fig. 1) indicates that the ulnar shaft became vestigial independently in elephant shrews, bats, and horses.

### Hand and fingers

**Figure 7:**
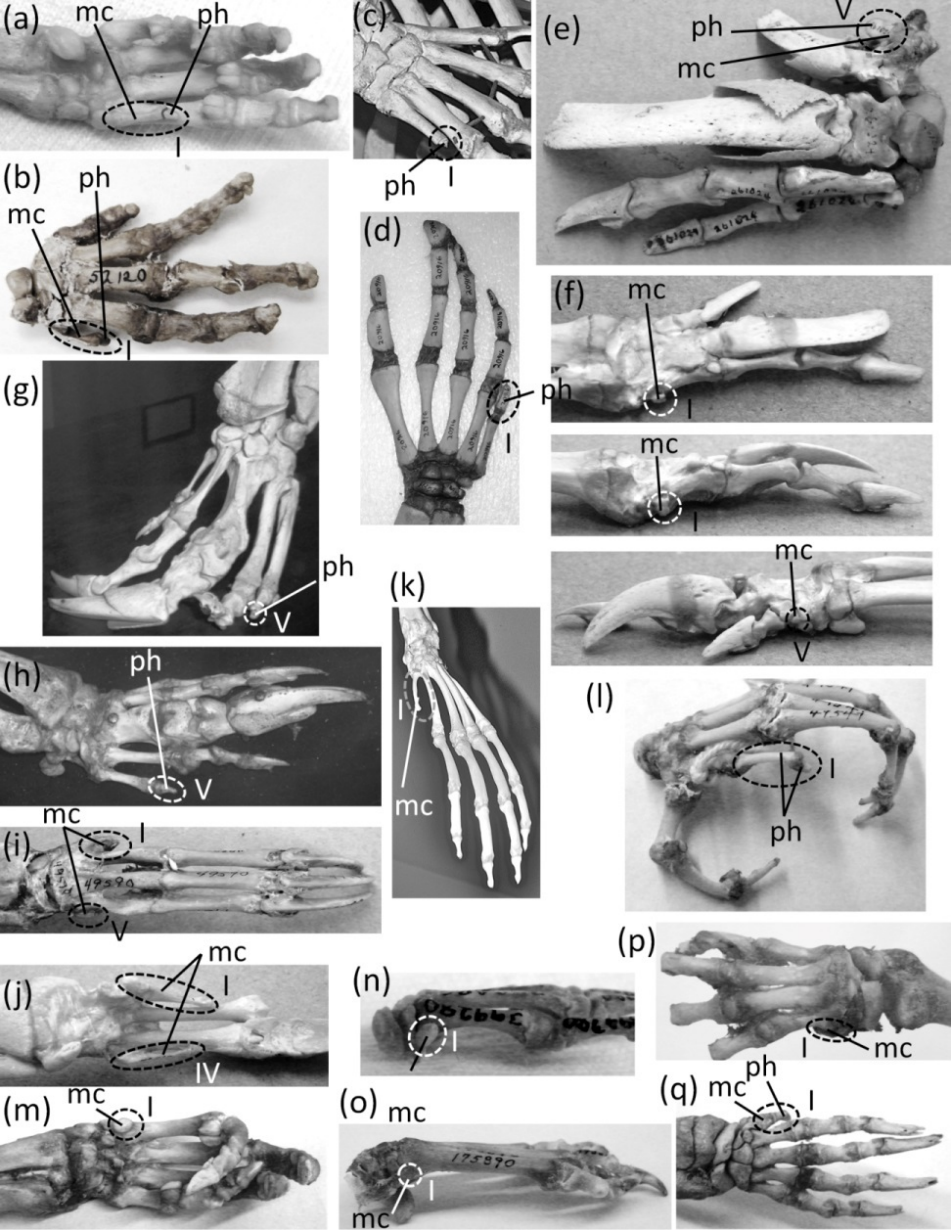

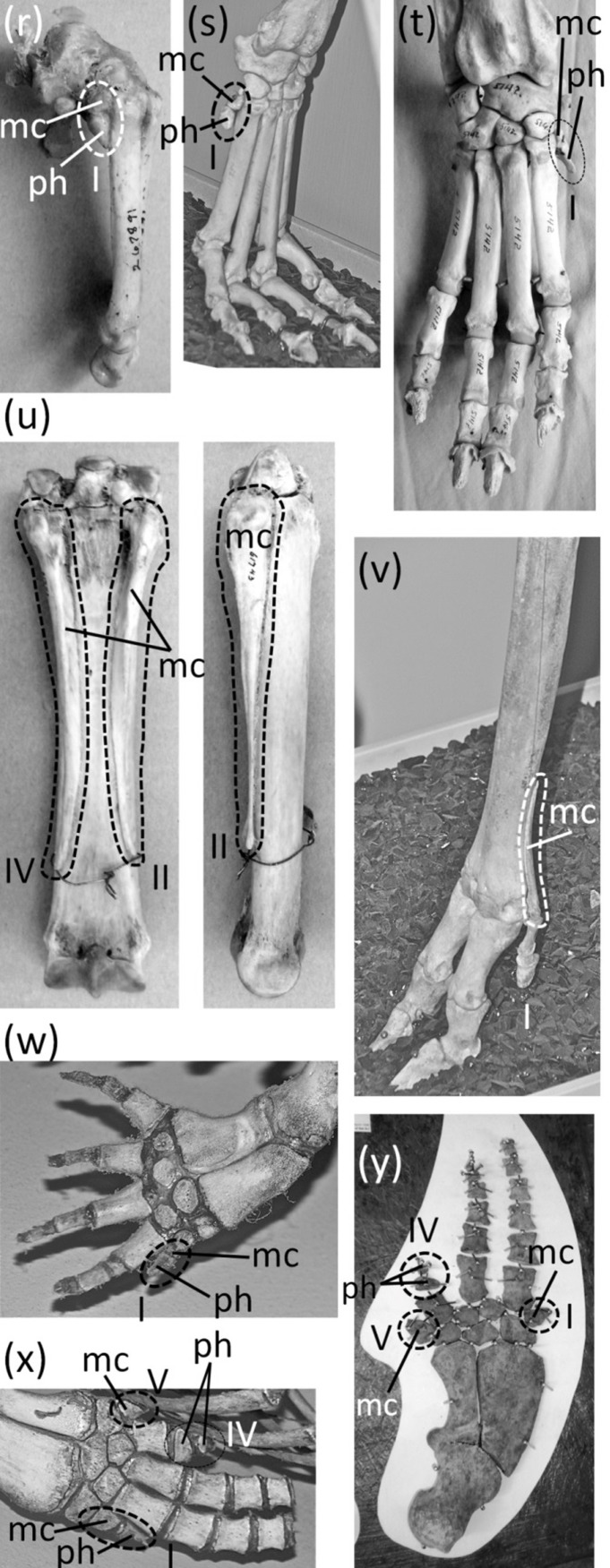
Vestigial fingers and metacarpals, circled with broken line. (A) *Procavia capensis* (rock hyrax), palmar view (B) *Dendrohyrax dorsalis* (western tree hyrax), dorsal view (C) *Dugong dugon* (dugong) (D) *Trichechus inunguis* (Amazonian manatee), dorsal view (E) *Priodontes maximus* (giant armadillo), dorsal view (F) *Tolypeutes matacus* (southern three-banded armadillo) in dorsal (top), medial (middle), and lateral (bottom) views (G) *Myrmecophaga tridactyla* (giant anteater), dorsal view (H) *Tamandua tetradactyla* (southern tamandua), dorsal view (I) *Bradypus variegatus* (brown-throated three-toed sloth), dorsal view (J) *Choloepus didactylus* (Darwin’s two-toed sloth), palmar view (K) *Ateles geoffroyi* (Geoffroy’s spider monkey) (L) *Perodicticus potto* (potto), dorsomedial view (M) *Hydrochoerus hydrochaerus* (capybara), palmar view (N) *Kerodon rupestris* (rock cavy), medial view (O) *Dolichotis patagonica* (Patagonian mara), medial view (P) *Lagostomus trichodactylus* (plains viscacha), dorsal view (Q) *Erethizon dorsatum* (North American porcupine), dorsal view (R) *Hyaena brunnea* (brown hyena), medial view (S) *Hyaena hyaena* (striped hyena) (T) *Crocuta crocuta* (spotted hyena), dorsal view (U) *Equus burchellii* (Burchell’s zebra) in palmar (left) and medial (right) views (V) *Odocoileus hemionus* (mule deer) (W) *Pontoporia blainvillei* (La Plata dolphin), dorsal view (X) *Delphinus delphis* (short-beaked common dolphin), palmar view (Y) *Tursiops truncatus* (common bottlenose dolphin), dorsal view.

Ancestrally, the mammalian hand has five digits with two phalanges in the thumb and three phalanges in each other finger, and a metacarpus in which all five metacarpals are of similar diameter (Fig. 2B). The metacarpals function as struts between the phalanges and the carpus. In numerous mammalian taxa one or more fingers have become vestigial (Fig. 7). In numerous others, one or more fingers are lost and the associated metacarpals are reduced to a vestigial state. We did not find examples of identifiably vestigial carpal bones.

In the marsupial *Chaeropus ecaudatus* (the recently-extinct pig-footed bandicoot) the first and fifth fingers and their metacarpals are lost, and the fourth finger and its metacarpal are vestigial. Together they are less than one-third the full length of the metacarpus (Flower, 1870).

In the Hyracoidea (hyraxes) the thumb is vestigial and is not externally visible. Its metacarpal is highly reduced in both length and diameter. It bears a single, miniscule phalanx (Flower, 1870) (Figs. 7A and 7B).

In the Sirenia the thumb is vestigial and usually retains only one phalanx. In *Dugong* (dugongs) the phalanx is reduced to a pebble-like nub (Fig. 7C). In *Trichechus* (manatees) the phalanx is relatively larger than in dugongs but is very reduced in length and diameter in comparison to the proximal phalanges of the other digits (Fig. 7D).

Vestigial fingers are abundant in the Xenarthra (armadillos, anteaters, and sloths). The fifth finger is vestigial in *Priodontes maximus* (giant armadillo). Its metacarpal is tiny, and it retains only one phalanx, which is reduced to a nub (Humphry, 1870) (Fig. 7E). In *Tolypeutes matacus* (southern three-banded armadillo) the first and fifth fingers are lost. Their metacarpals are vestigial; each is reduced to a tiny, pebble-like, transversely flattened bone (Fig. 7F). In *Dasypus novemcinctus* (nine-banded armadillo) the fifth finger is vestigial; it is present only as a single, miniscule, grain-shaped phalanx. In *Myrmecophaga tridactyla* (giant anteater) the fifth finger retains two phalanges but has lost the third, and the second is reduced to a nub and can therefore be considered vestigial (Fig. 7G). In *Tamandua* (lesser anteaters) the fifth finger is vestigial. It is reduced to a single phalanx that is but a nub (Fig. 7H). In *Cyclopes didactylus* (silky anteater), the phalanges of the thumb and fifth finger are lost, as is the fifth metacarpal, and the first metacarpal is vestigial. It is reduced to a short, subquadrangular, transversely flattened bone. In *Bradypus* (three-toed sloths) the first and fifth fingers are lost. The corresponding metacarpals are reduced to a vestigial state, remaining only as small, hook-shaped bones (Humphry, 1870) that may be coossified with the neighboring metacarpals (Fig. 7I). In *Choloepus* (two-toed sloths) the first, fourth, and fifth fingers are lost, as is the fifth metacarpal. The first and fourth metacarpals are vestigial; each is approximately half the length of the neighboring metacarpal and is very reduced in diameter (Humphry, 1870) (Fig. 7J).

In *Ateles* (spider monkeys), *Brachyteles* (woolly monkeys), and *Colobus* (colobus) the thumb is vestigial. Its metacarpal is reduced in diameter and length (Fig. 7K). Some specimens retain a single phalanx that is reduced to a nub, while in others the thumb lacks phalanges (Tague, 1997; Tague, 2002).

In *Perodicticus potto* (potto) and *Arctocebus* (angwantibos), the African members of the primate family Lorisidae, the second finger is vestigial, although the thumb is fully expressed. The second metacarpal is reduced in length, its proximal phalanx is reduced in length and diameter, its middle phalanx is reduced to a nub, and its distal phalanx is lost (Fig. 7L). In the fleshed-out animal, the second finger is reduced to a short stump in *Arctocebus*. In *P. potto* it is further reduced and exists as a mere lump at the edge of the palm.

**Figure 8:**
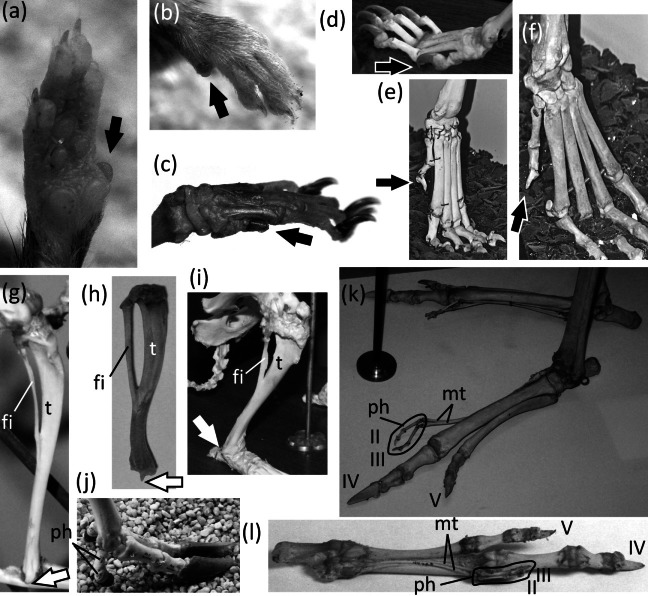
Reduced structures that fail to satisfy one or more of the three criteria for vestigiality. Thumb of certain rodents (A–C), thumb of certain carnivores (dewclaw) (D–F), fibula that is fused to the tibia (G–I), second and fifth toes of deer (J), and syndactylous second and third toes of marsupials (K–L). Black arrows indicate thumbs. White arrows indicate the distal end of the fibula. (A) *Mus musculus* (house mouse), palmar view of right hand (B) Same individual as in *a*, left hand in dorsal view; note the primate-like thumbnail (C) *Tamias striatus* (eastern chipmunk) (D) *Felis catus* (domestic cat) (E) *Canis aureus* (golden jackal) (F) *Proteles cristata* (aardwolf) (G) *Cephalopachus bancanus* (Horsfield’s tarsier) (H) *Ondatra zibethicus* (muskrat) (I) *Oryctolagus cuniculus* (domestic rabbit) (J) *Odocoileus vriginianus* (white-tailed deer) (K) *Macropus canguru* (great gray kangaroo) (L) *Aepyprymnus rufescens* (rufous rat-kangaroo), plantar view.

Reduction of the thumb (Fig. 8) is common in rodents. In many cases it is miniscule in comparison to the other fingers (Figs. 8A and 8B). This is common in the Muroidea (rats, mice, and kin), Dipodidae (jerboas, jumping mice, and kin), Gliridae (dormice), Heteromyidae (kangaroo rats and kin), Octodontidae (degus and kin), and Sciuridae (squirrels) (Kingdon, 1974; Kingdon, 1997; Garbutt, 1999; and P Senter, pers. obs., 2012–2014). It is also the case in *Chinchilla* (chinchillas). In many such cases, the claw on the thumb is not pointed at the tip but is flattened into a shape that resembles a primate’s nail (Figs. 8A–8C). Because the terminal phalanx is therefore an ungual, the digit does not satisfy the second criterion for vestigiality. Also, in such cases the tip of the thumb is used in opposition to the second finger, to grasp objects, as P Senter has personally observed (pers. obs. 2014, 2015) in the chinchilla (*Chinchilla lanigera*) and the degu (*Octodon degus*); the thumb therefore does not satisfy the third criterion for vestigiality.

In several other rodent families are a plethora of cases in which the thumb is lost or is so reduced that it does not protrude externally and satisfies all three criteria for vestigiality. In the squirrel genus *Sciurus* is an ambiguous case: the thumb is tipped with a claw, but only the claw protrudes externally. Future studies will be necessary to determine what function, if any, this thumb claw serves.

The thumb is lost in the Caviidae (cavies, capybaras, and kin), and the metacarpal is vestigial. It remains only as a tiny, ovoid bone no larger than a distal carpal and usually much smaller (Figs. 7M–7O). In the Chinchillidae the thumb is lost in *Lagidium* (mountain viscachas) and *Lagostomus trichodactylus* (plains viscacha). In the latter two the first metacarpal is vestigial; it is a tiny, transversely flattened ovoid (Fig. 7P). In *Erethizon dorsatum* (North American porcupine) and *Coendou* (prehensile-tailed porcupines) the thumb is vestigial. Its metacarpal is highly reduced, and it retains only one highly reduced phalanx with a variable shape (Fig. 7Q).

**Figure 9:**
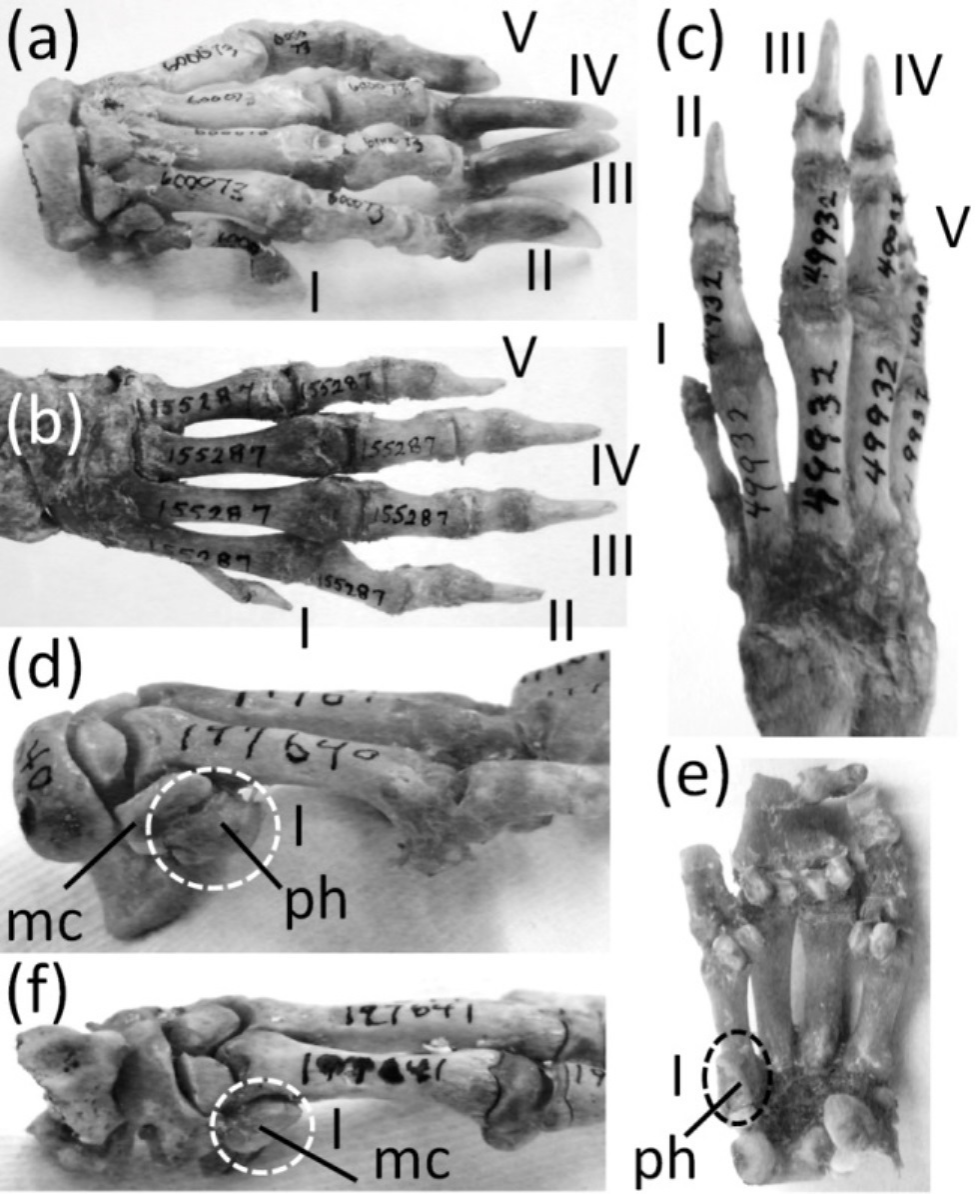
Various levels of thumb expression in *Hystrix* (Old World porcupines), with vestigial structures circled with broken line. (A) *H. indica* (Indian porcupine), with undreduced thumb, dorsal view; (B) *H. sumatrae* (Sumatran porcupine), with thumb that is reduced in diameter, dorsal view; (C) *H. javanica* (Sunda porcupine), with thumb that is reduced in diameter, dorsal view; (D) *H. crassispinis* (thick-skinned porcupine), medial view, with vestigial thumb; (E) *H. crassispinis*, palmar view; (F) *H. brachyura* (Malayan porcupine), in which the thumb is lost, in medial view.

The genus *Hystrix*, a member of Hystricidae (Old World porcupines) is unusual in that different species of one genus exhibit different degrees of thumb reduction (Fig. 9). In *H. indica* (the Indian porcupine) the thumb is fully expressed and robust. In *H. sumatrae* (the Sumatran porcupine), *H. javanica* (the Sunda porcupine) and *H. cristata* (the crested porcupine) the thumb is reduced relative to its state in *H. indica*, but it is not vestigial. In *H. crassispinis* (the thick-skinned porcupine) the thumb is vestigial. It retains only one phalanx, which is no larger than the highly reduced metacarpal that is typical for the genus. In *H. brachyura* (the Malayan porcupine) the thumb is lost, leaving only its reduced metacarpal.

In the Canidae and Felidae the thumb (Figs. 8D and 8E) is called the dewclaw. It is shorter than the other digits and does not contact the ground. Some authors consider it vestigial (e.g., Eldredge, 2007), but it is not reduced enough to satisfy the first criterion for vestigiality. Also, it retains a claw and therefore does not satisfy the second criterion. Nor does it satisfy the third criterion, because it retains the typical function of a finger with a sharp, curved ungual and claw: prehension. Its use is an important part of prey capture in felids (Londei, 2000), and we have personally observed that domestic dogs use the dewclaw to snag and maintain a grip on objects. Its shortening is therefore not the reduction of an unused organ. We suggest that the functional advantage of the shortening is to keep the claw sharp by preventing wear that would result from contact with the ground. Indeed, according to P Senter (pers. obs., 2005–2006), the canid dewclaw has a sharper, less worn tip than the other claws and is more effective at puncturing and maintaining prehension. An analogy can therefore be made between the dewclaw and the second toe of dromaeosaurid and troodontid dinosaurs, which was also held clear of the ground (Senter, 2009), was used to puncture (Fowler et al., 2011), and had a function that differed from those of the other digits, as shown by its difference in length and in claw curvature.

A vestigial dewclaw is present in *Hyaena* (striped hyena and brown hyena) and *Crocuta* (spotted hyena). In both, the metacarpal is reduced to a small block, the distal phalanx is lost, and the proximal phalanx is reduced. The proximal phalanx is a small, shapeless lump in *Hyaena* and a tiny spike in *Crocuta* (Figs. 7R–7T). The hyaenid *Proteles cristata* (aardwolf) has an unreduced dewclaw (Fig. 8F).

In the Perissodactyla the first finger and its metacarpal are lost. In *Equus* (horses) the second, fourth, and fifth fingers are also lost, as are the first and fifth metacarpals. The second and fourth metacarpals are vestigial. They remain as thin splints that taper to a point without reaching the distal end of the metacarpus (Fig. 7U).

In the Cetartidoactyla the first finger is lost, and in ruminant cetartiodactyls the second and fifth fingers are reduced (Tragulidae [chevrotains] and Cervidae [deer]) or lost (Antilocapridae [pronghorn], and Giraffidae [giraffes and okapi]). The reduced second and fifth fingers of deer are called dewclaws (McBride, 2001; Elbroch, 2003), and some authors consider them vestigial (e.g., McBride, 2001). However, they bear hooves and therefore do not meet the second criterion for vestigiality. Nor do they meet the third criterion for vestigiality, because during fast locomotion they make sufficient contact with the ground (Elbroch, 2003) to exhibit a major function of digits: bodily support.

In the Cervidae (deer) the second and fifth fingers are not vestigial according to our criteria. However, the second and fifth metacarpals of Cervinae (Old World deer) are vestigial; they are reduced to proximal splints that resemble the vestigial metacarpals of horses (Geist, 1998). In the second and fifth metacarpals of Capreolinae (New World deer) the proximal end is lost, leaving only the distal end, which articulates with the proximal phalanx. The shaft of each of these metacarpals is vestigial and is reduced to a small splint (Geist, 1998) (Fig. 7V).

In the Bovidae (cattle, antelope, sheep, and goats) the second and fifth fingers have only one or two phalanges apiece, and these do not articulate with the rest of the skeleton. However, they bear hooves and therefore do not meet the second criterion for vestigiality. When present, the fifth metacarpal is vestigial. It remains only as a tiny, proximal splint (Nickel et al., 1986).

Vestigial fingers are common in the Odontoceti (toothed whales). In odontocetes other than Physeteroidea (sperm whales and kin) the thumb is vestigial (Figs. 7W–7Y). It typically either retains only a single, pebble-like phalanx, or just a metacarpal (Van Beneden & Gervais, 1879; Cooper et al., 2007). In a few species there are some individuals that have two thumb phalanges, but their conspecifics have only one phalanx or none (Cooper et al., 2007). In several odontocete clades, the fifth finger is also vestigial, retaining one or two pebble-like phalanges or just a reduced metacarpal. Such is the case in *Inia* (New World river dolphins), *Phocoena* (porpoises), and the delphinid subfamilies Delphininae (dolphins) and Globicephalinae (pilot whales and kin) (Van Beneden & Gervais, 1879; Cooper et al., 2007). In the latter two clades the fourth finger is also reduced enough to consider vestigial. It usually retains only two phalanges, and only the proximal phalanx has the typical form of an odontocete phalanx instead of being reduced to a tiny, pebble-shaped bone (Van Beneden & Gervais, 1879) (Figs. 7X and 7Y).

In addition to the first finger and its metacarpal, the fifth finger and its metacarpal are also lost in the Rhinocerotidae (rhinoceroses). Flower (1870) identified a small bone in the wrist of *Dicerorhinus sumatrensis* (Sumatran rhinoceros) as a vestigial fifth metacarpal, but it is more likely a sesamoid. It does not articulate with the lateral surface of the fourth metacarpal or the lateral surface of the hamate carpal, as would be expected of a fifth metacarpal. Rather, it is on the palmar surface of the hamate. We found the homologous bone in the wrist of a specimen of *Rhinoceros sondaicus* (Javan rhinoceros) and in photos, supplied by the Museum of Comparative Zoology, of articulated hands of two specimens of *R. unicornis* (Indian rhinoceros). The bone is small and rounded, is on the palmar side of the hamate, and does not articulate with the lateral surface of the hamate or the fourth metacarpal. These are characteristics that are consistent with a sesamoid but not with a vestigial fifth metacarpal.

The phylogenetic distribution of character states (Fig. 1) indicates that the thumb independently became vestigial at least seven times in the Mammalia: in the Sirenia, Hyracoidea, *Lagidium* + *Lagostomus*, Erethizontidae, *Hystrix crassipes*, *Crocuta* + *Hyaena*, and Odontoceti. In the preceding sentence and below, the phrase “at least” expresses uncertainty as to whether a skeletal structure became vestigial before its loss in the taxa that have lost it. The thumb was independently lost eight times: in *Chaeropus*, Tubulidentata, *Ateles* + *Brachyteles*, *Colobus*, Caviidae, Perissodactyla, Camelidae, and Ruminantia. The second finger became vestigial once: in *Arctocebus* + *Perodicticus*. It was lost independently four times: in the Antilocapridae, Giraffidae, Camelidae, and Equidae. The fourth finger became vestigial in *Chaeropus*, and is not vestigial in any other Recent mammal. It was independently lost twice: in *Choloepus* and *Equus*. The fifth finger became vestigial at least seven times: in *Priodontes*, *Dasypus*, *Tamandua*, Globicephalinae, Delphininae, *Phocoena*, and *Inia*. It was independently lost ten times: in *Chaeropus*, *Cyclopes*, *Tolypeutes*, *Equus*, Rhinocerotidae, Camelidae, Antilocapridae, Giraffidae, *Bradypus*, and *Choloepus*. Although *Choloepus* is the closest living relative to *Bradypus*, it must have lost its fifth finger independently, because the two genera are in different families, and some extinct members of the Megalonychidae (which includes *Choloepus*) retained a vestige of the fifth finger (P Senter, pers. obs., 2008).

The first metacarpal became vestigial independently at least eight times in the Mammalia: in *Ateles* + *Brachyteles*, *Colobus*, *Tolypeutes*, *Cyclopes*, Caviidae, *Equus*, *Bradypus*, and *Choloepus* (some extinct megalonychids retained a fully expressed first metacarpal). The first metacarpal was independently lost four times: in Tubulidentata, Perissodactyla, Camelidae, and Ruminantia. The second metacarpal became vestigial independently at least twice: in *Equus* and Cervidae. It was lost independently lost four times: in Camelidae, Antilocapridae, Giraffidae, and Bovidae. The fourth metacarpal became vestigial independently three times: in *Chaeropus*, *Choloepus*, and *Equus*. The fifth metacarpal became vestigial independently at least three times: in *Tolypeutues*, *Bradypus*, and Bovidae + Cervidae. It was independently lost eight times: in *Chaeropus*, *Choloepus*, *Cyclopes*, *Equus*, Rhinocerotidae, Camelidae, Antilocapridae, and Giraffidae.

### Pelvic girdle

Ancestrally, the mammalian pelvic girdle consists of three bones: the ilium, ischium, and pubis (Figs. 2E–2G). All three bones contribute to the acetabulum (hip socket). The ilium is attached to the vertebral column, and via this attachment the hindlimb propels the entire vertebral column during locomotion. The vertebrae that contact the ilium are fused to a few more vertebrae posterior to them. Together this series of fused vertebrae is called the sacrum (Figs. 2E and 2F). The left and right pubes are ventral in location and meet in the midline at a symphysis. The left and right ischia extend posteriorly and do not meet each other. The pubis and ischium surround an opening called the obturator foramen. In most mammals, by adulthood the three bones of the pelvic girdle have fused together to form a single bone called the coxal bone or innominate.

**Figure 10:**
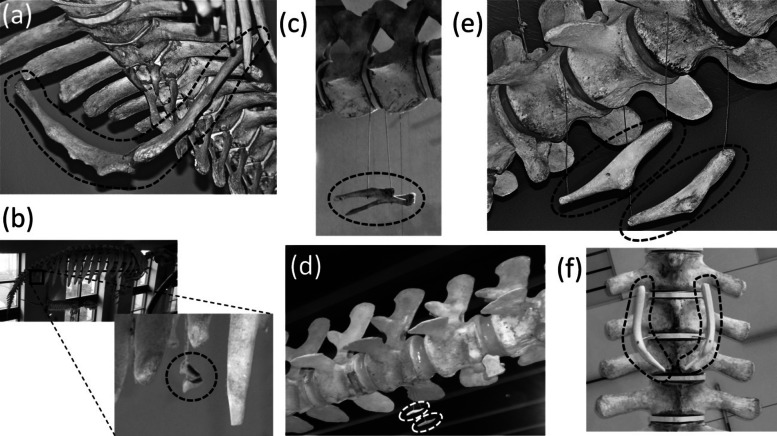
Vestigial pelvic girdles, circled with broken line. (A) *Dugong dugon* (dugong) (B) *Trichechus manatus* (West Indian manatee) (C) *Physeter catodon* (sperm whale) (D) *Delphinapterus leucas* (beluga whale) (E) *Eschrichtius robustus* (gray whale) (F) *Eubalaena glacialis* (North Atlantic right whale).

The pelvic girdle is vestigial in Sirenia. It is extremely reduced in size and has lost contact with the vertebral column (Figs. 10A and 10B). Abel’s (1908) comparison of the pelvic girdles of extant and fossil Sirenia shows that the pelvic girdle of the dugong (*Dugong dugon*) retains the acetabular region, which is where the ilium, ischium, and pubis converge. Therefore, none of the three bones is lost. In the dugong the pubis is highly reduced, and the pelvic girdle consists mainly of the ilium and ischium (Abel, 1908). According to Abel (1908), in *Trichechus* (manatees), the pubis and ilium are both lost or reduced almost unto loss, leaving only the ischium, which retains its original shape.

The pelvic girdle is vestigial in Cetacea (whales). It is extremely reduced in size, has lost contact with the vertebral column, and has lost a distinct acetabulum (Figs. 10C–10F). In Mysticeti the pelvic girdle is usually shaped like a very shallow “V,” with an anterior process meeting a posterior process at an obtuse angle, and with a third, much shorter process projecting from the point of union between the two main processes, slightly extending the point of the “V” (Howell, 1930; Lönneberg, 1910; Arvy, 1976) (Figs. 10E and 10F). In Odontoceti the pelvic girdle is typically not V-shaped but is a straight or slightly curved rod (Howell, 1930; Lönneberg, 1910; Arvy, 1976) (Figs. 10C and 10D).

Previous authors have disagreed as to whether the cetacean pelvic girdle retains the ischium alone (Struthers, 1881; Simões-Lopes & Gutstein, 2004), the ischium and ilium (Abel, 1908; Lönneberg, 1910; Schulte, 1916; Howell, 1930), or the ischium, ilium, and pubis (Gol’din, 2014). Its connections with soft anatomy indicate that much of the girdle is the ischium (Struthers, 1881; Simões-Lopes & Gutstein, 2004). However, the presence of the acetabulum in mysticetes (Struthers, 1881; Fordyce et al., 2000; Gol’din, 2014) suggests that all three bones are present, at least in reduced form, in the mysticete pelvic girdle. No part of the odontocete pelvic girdle bears evidence that it represents the pubis or ilium (Simões-Lopes & Gutstein, 2004), and its simple rodlike shape suggests that one tine (containing the vestigial pubis and ilium) of the mysticete “V” has been lost, leaving only the ischium. In the reduced pelvic girdles of *Basilosaurus isis* and *Chrysocetus healyorum*, members of the extinct whale family Basilosauridae from the Eocene Epoch, the ischium and ilium are more highly reduced than the pubis, which meets its counterpart at a midline symphysis (Gingerich, Smith & Simons, 1990; Uhen & Gingerich, 2001). Apparently, then, in the evolution of the cetacean pelvis, reduction of the ilium and ischium occurred first, followed by the reduction of the pubis and ilium and subsequently their loss in odontocetes. The cetacean pelvic girdle is certainly not an abdominal bone such as marsupials have, as one author has suggested (Arvy, 1976; Arvy, 1979), because it retains a muscular or ligamentous connection to the femur in specimens that retain vestigial hindlimbs (Struthers, 1881; Hosokawa, 1951; Ogawa & Kamiya, 1957), and because the soft tissues that attach to it are those that typically attach to an ischium (Struthers, 1881; Simões-Lopes & Gutstein, 2004).

The phylogenetic distribution of character states (Fig. 1) shows that the pelvis became vestigial twice independently: in the Sirenia and Cetacea.

### Femur, tibia and fibula

Ancestrally, the mammalian hindlimb includes a single bone in the thigh (the femur) and two in the shank or crus (the tibia and fibula, with the fibula the more lateral of the two). The proximal end of the tibia articulates with the femur, and the proximal end of the fibula articulates with a lateral shelf of the tibia. Both the tibia and the fibula articulate distally with the tarsus (Figs. 2C and 2D). The fibula functions as a strut between the tarsus and the proximal tibia.

The shaft of the fibula is fused to the tibia in many small mammals, including Macroscelidea (elephant shrews), Tarsiidae (tarsiers), Eulipotyphla (shrews, moles, hedgehogs, and kin), Lagomorpha (rabbits and pikas), and many rodents. In some cases only the distal half of the fibular shaft is fused to the tibia, but in others only a small, proximal portion of the fibula is free of the tibia (Figs. 8G–8I). This yields the illusion that most of the fibula has been lost, in which case the remaining portion could be considered vestigial. However, close inspection shows that even in cases with extreme amounts of fusion, the fibula is present for its full length and is a strut between the tarsus and the proximal tibia. It therefore does not satisfy the first or third criterion for vestigiality.

**Figure 11:**
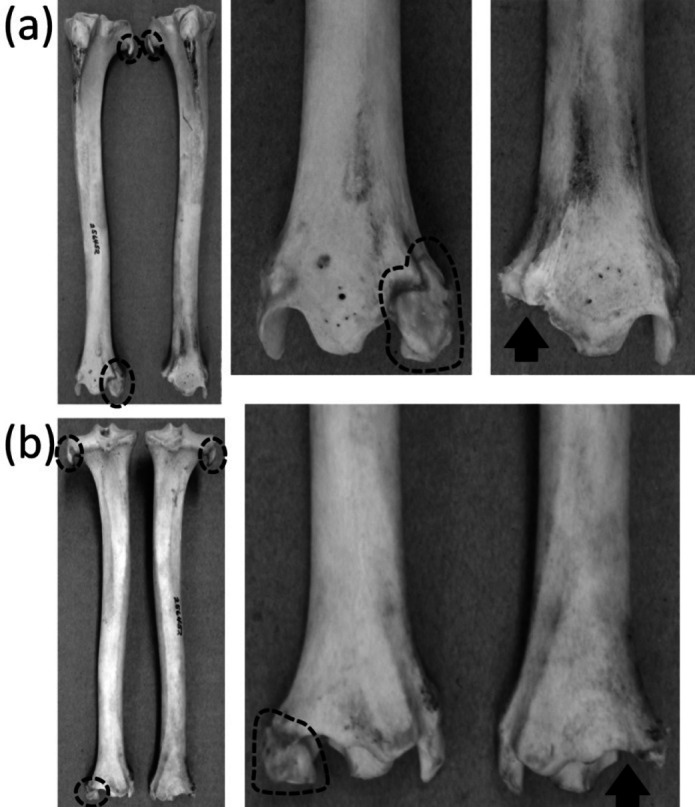
Crurae (tibiae + fibulae) of *Antilocapra americana* (pronghorn), with the specimen’s left crus on the viewer’s left and the specimen’s right crus on the viewer’s right. Parts of vestigial fibulae are circled with broken line. Note that the distal fibula is missing from the right crus, on which an arrow indicates the socket for the distal fibula. (A) Anterior view (B) Posterior view.

An unambiguously vestigial fibula is present in three extant ungulate taxa: Camelidae (camels and kin), Pecora (ruminants other than chevrotains), and *Equus* (horses). In Camelidae the shaft and proximal end of the fibula are lost. All that remains is a distal vestige: a block of bone called the malleolar bone or os malleolare, which fits into a cleft in the tibia and articulates with the two proximal tarsal bones (Flower, 1870). In Pecora the shaft of the fibula is replaced by a ligament, and its proximal and distal extremities remain as vestiges. The proximal vestige, all that remains of the head of the fibula, is a small spike that is fused to the lateral condyle of the tibia. The distal vestige is a malleolar bone resembling that of camelids (Nickel et al., 1986) (Fig. 11). In *Equus* the distal half of the fibular shaft is lost. The proximal vestige of the tibia includes the head and a thin rod that represents the remainder of the fibular shaft. The distal vestige is similar to that of camelids and pecorans but is fused to the tibia (Nickel et al., 1986).

Hindlimb buds appear in the embryos of both mysticete and odontocete whales (Ogawa, 1953; Bejder & Hall, 2002). In odontocetes the hindlimb buds regress and limbs are not formed, except in occasional atavistic cases (Ogawa & Kamiya, 1957; Ohsumi, 1965). In mysticetes, vestigial hindlimbs are often present, with much individual variation in morphology (Struthers, 1881; Hosokawa, 1951; Gol’din, 2014). Typically, the bowhead whale (*Balaena mysticetus*) retains the femur and tibia; the humpback (*Megaptera novaeangliae*) and fin whale (*Balaenoptera physalus*) retain only the femur; the minke whale (*Balaenoptera bonaerensis*) retains the femur in about one-third of individuals; and the hindlimb is absent in the sei whale (*Balaenoptera borealis*) (Struthers, 1881; Hosokawa, 1951; Omura, 1980). Occasional atavistic specimens retain the more distal elements. For example, Andrews (1929) described a humpback whale with an ossified tibia and metatarsal and a cartilaginous femur and tarsus.

In the extant Sirenia there is usually no hindlimb. However, an example of an atavistic, diminutive femur has been described in an example of *Trichechus manatus* (West Indian manatee) (Abel, 1908). It is tiny enough to consider vestigial.

The phylogenetic distribution of character states (Fig. 1) shows that the femur was independently lost three times: in the Sirenia, Odontoceti, and *Balaenoptera borealis*. The crus was independently lost three times, in the Sirenia, Odontoceti, and Balaenopteridae. The fibula became vestigial independently at least three times: in the Camelidae, Pecora, and *Equus*.

### Foot and toes

**Figure 12:**
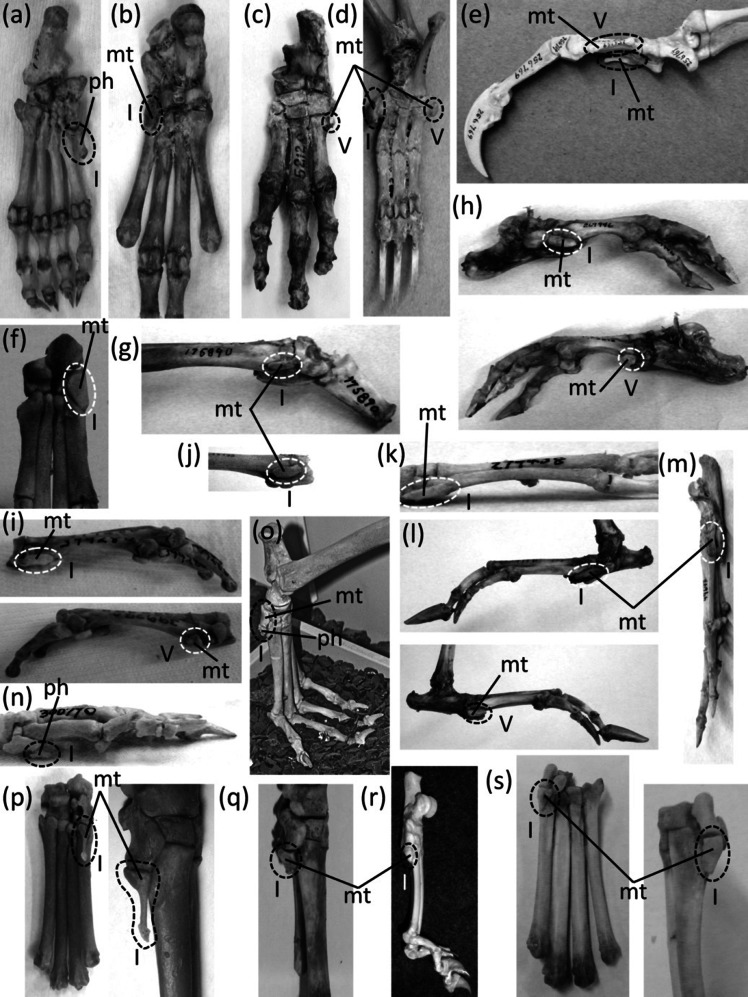

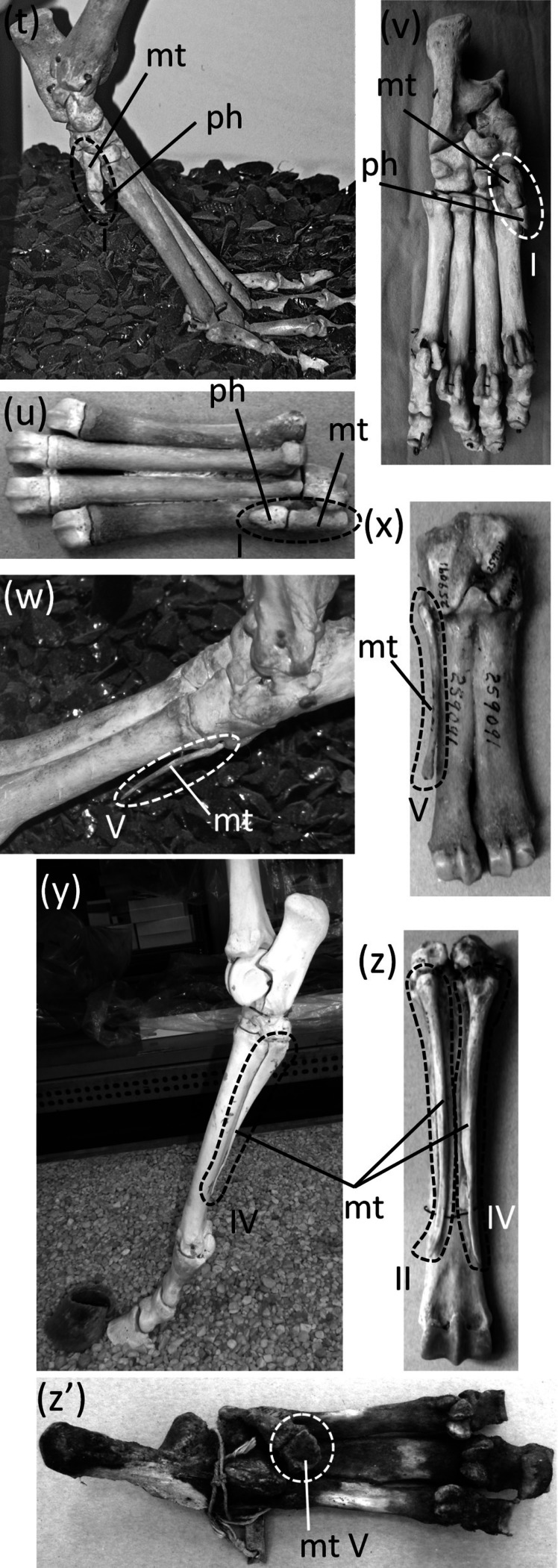
Vestigial toes and metatarsals. (A) *Sarcophilus harrisii* (Tasmanian devil), plantar view (B) *Thylacinus cynocephalus* (Tasmanian wolf), plantar view (C) *Dendrohyrax dorsalis* (western tree hyrax), dorsal view (D) *Bradypus variegatus* (brown-throated three-toed sloth), plantar view (E) *Choloepus didactylus* (Darwin’s two-toed sloth), lateral view (F) *Sylvilagus floridanus* (eastern cottontail), plantar view (G) *Dolichotis patagonica* (Patagonian mara), medial view (H) *Hydrochoerus hydrochaerus* (capybara), medial (above) and lateral (below) views (I) *Kerodon rupestris* (rock cavy), medial (above) and lateral (below) views (J) *Dasyprocta leporina* (red-rumped agouti), medial view (K) *Chinchilla chinchilla* (short-tailed chinchilla), medial view (L) *Lagostomus trichodactylus* (plains viscacha), medial (above) and lateral (below) views (M) *Thryonomys gregorianus* (lesser cane rat), medial view (N) *Pedetes capensis* (South African springhare), medial view (O) *Canis familiaris* (domestic dog: collie) (P) *Canis latrans* (coyote), plantar (left) and medial (right), views (Q) *Vulpes vulpes* (red fox), medial view (R) *Felis catus* (domestic cat), medial view (S) *Lynx rufus* (bobcat), plantar (left) and medial (right) views (T) *Proteles cristata* (aardwolf), medial view (U) *Hyaena hyaena* (striped hyena), plantar view (V) *Crocuta crocuta* (spotted hyena), plantar view (W) *Pecari tajacu* (collared peccary), dorsolateral view (X) *Tayassu pecari* (white-lipped peccary), plantar view (Y) *Equus caballus* (domestic horse), lateral view (Z) *Equus burchellii* (Burchell’s zebra), plantar view (Z’) *Tapir terrestris* (Brazilian tapir).

Ancestrally, the mammalian foot has five digits with two phalanges in the first toe and three phalanges in each other toe, and a metatarsus in which all five metatarsals are of similar diameter (Fig. 2C). The metatarsals function as struts between the phalanges and the tarsus. In numerous mammalian taxa one or more toes have become vestigial (Fig. 12). In numerous others, one or more toes are lost and the associated metatarsals are reduced to a vestigial state. We did not find examples of identifiably vestigial tarsal bones.

In marsupials of the orders Peramelemorphia (bandicoots and bilbies) and Diprotodontia (wombats, possums, kangaroos, and kin), the second and third toes are syndactylous. The skeletons of the two digits share a common sheath of soft tissue, so that the two toes are separate only at the last phalanx, which bears the claw. The two toes are therefore functionally a single toe with two claws. The metatarsals and phalanges of the two toes are usually half or less the diameter of those of the fourth toe, and their reduction in size makes them appear vestigial (Figs. 8K and 8L). However, the two digits do not meet the second criterion for vestigiality, because they bear claws. They also fail to meet the third criterion for vestigiality, because together they functionally constitute a single toe that is used as a toe.

*Chaeropus ecaudatus* (pig-footed bandicoot) is an exception to the above rule. Its second, third, and fifth toes fit all three criteria for vestigiality (Flower, 1870). The foot of *C. ecaudatus* is functionally monodactyl and uses only the fourth toe for support.

The first toe is reduced in some members of the marsupial family Dasyuridae. It is reduced enough to fit all three criteria for vestigiality in *Antechinomys laniger* (the kultarr) (Szalay, 1994), *Dasyuroides byrnei* (the kowari), and *Sarcophilus* (Tasmanian devils). In all three cases it is represented only by a very short metatarsal and a single phalanx shaped like a small spike (Fig. 12A).

In *Thylacinus cynocephalus*, the recently-extinct thylacine or Tasmanian wolf, the first toe is lost, and the first metatarsal is vestigial. It is a flattened oval, not much longer than the transverse width of one of the other metatarsals (Fig. 12B).

In the Hyracoidea the first and fifth toes are lost, as is the first metatarsal. The fifth metatarsal is vestigial. It is tiny and transversely flattened (Fig. 12C).

In *Bradypus* (three-toed sloths) and *Choloepus* (two-toed sloths) the first and fifth toes are lost, and the first and fifth metatarsals are reduced to a vestigial state. In *Bradypus* these two metatarsals remain only as small, hook-shaped bones (Humphry, 1870) that are coossified with the neighboring metatarsals (Fig. 12D). In *Choloepus* the first and fifth metatarsal are each little more than half the length of the neighboring metatarsal (Flower, 1870); these vestigial metatarsals are transversely flattened and lack a distal articulating surface (Fig. 12E).

In the Lagomorpha the first toe is lost. Its metatarsal is vestigial in Leporidae (rabbits) (Fig. 12F) and absent in Ochotonidae (pikas).

In the Caviidae the first and fifth toes are lost, and their metatarsals are vestigial (Figs. 12G–12I). Previous authors (e.g., Mivart & Murie, 1866; Cooper & Schiller, 1975) have identified each of these two vestiges as a sesamoid, which is a bone that ossifies inside a tendon or ligament. However, several lines of evidence show that these two bones are not sesamoids but are the first and fifth metatarsals. Most importantly, as shown in Cooper & Schiller’s (1975) illustrations, neither of the two bones is within a tendon or ligament. Secondly, the two bones are in the locations of the proximal ends of the first and fifth metatarsals of other rodents, and they exhibit the articulations with neighboring bones that the first and fifth metatarsals of other rodents do. Furthermore, the vestigial first metatarsal strongly resembles its counterpart in other rodents, and the proximal end of the vestigial fifth metatarsal serves as the insertion of the peroneus brevis muscle (Cooper & Schiller, 1975), which inserts on the proximal end of the fifth metatarsal in other mammals (Parsons, 1898; Meehan, 1992; Marieb, Mallatt & Wilhelm, 2005; Budras et al., 2007), including other rodents (Parsons, 1898; Hebel & Stromberg, 1986). In all caviid genera, the vestigial first and fifth metatarsals are transversely flattened. The first metatarsal is a proximodistally elongate ovoid, and the fifth is near-circular in shape in lateral view.

In the Dasyproctidae (agoutis and acouchis) the first and fifth toes are lost, as is the fifth metatarsal. The first metatarsal is vestigial (Fig. 12J) and resembles that in the Caviidae and Chinchillidae.

In the Chinchillidae (chinchillas and viscachas) the first toe is lost and its metatarsal is vestigial. It is a transversely flattened, proximodistally elongate ovoid (Figs. 12K and 12L). Of the three genera in this family, the fifth toe is present and fully expressed in two: *Chinchilla* (chinchillas) and *Lagidium* (mountain viscachas). In the remaining species, *Lagostomus trichodactylus* (the plains viscacha), the fifth toe is absent and its metatarsal is vestigial; it resembles the first metatarsal but is proximodistally shorter (Fig. 12L).

In the Pedetidae (springhares) the first toe is lost, and its metatarsal is vestigial. As in other rodents with a vestigial first metatarsal, it is transversely flattened and proximodistally elongate, and its distal margin is rounded. However, unlike the case in other rodents, it tapers to a point proximally (Fig. 12M).

In the Thryonomyidae (cane rats) the first toe is vestigial. It retains only one phalanx, which is less than half the size of the highly reduced metatarsal and is shaped like a small spike (Fig. 12N).

In *Dipodomys* (kangaroo rats) and *Jaculus* (African jerboas), members of the Dipodidae, the first toe is lost, and its metatarsal is vestigial; it is reduced to a tiny, proximal sliver (Howell, 1932). In other members of the Dipodidae all five toes are fully expressed.

Most members of the Carnivora retain all five toes. Exceptions are the families Canidae, Felidae, and Hyaenidae. In the Canidae the first toe is vestigial. Its metatarsal is tiny, and its toe has only one phalanx, which is reduced to a small spike or nubbin (Figs. 12O–12Q). In the Felidae the first toe is lost and its metatarsal is vestigial (Flower, 1870) (Figs. 12R and 12S). In the Hyaenidae, as in the Canidae, the first toe is vestigial. Its metatarsal is tiny, and its toe has only one phalanx, which is reduced to a small spike or nubbin (Figs. 12T–12V).

In peccaries (Tayassuidae) the fifth toe is lost, and its metatarsal is retained but vestigial. It is a transversely flattened, proximal splint that is shorter than the other metatarsals (Figs. 12W and 12X).

The second and fifth toes are reduced in the Tragulidae (chevrotains), Bovidae (cattle, antelope, sheep, and goats), and Cervidae (deer), but they do not meet our criteria for vestigiality, because they bear hooves (Fig. 8J). The second and fifth metatarsals are fully expressed in the Tragulidae. It was once thought that these two metatarsals are absent in other ruminants (Flower, 1870), but they are often present and fused to the rest of the metatarsus. When present, each remains only as a proximal sliver that fits the criteria for vestigiality. Vestigial second metatarsals are common in the Bovidae (cattle and kin), Capreolinae (New World deer), and Giraffidae (giraffe and okapi); they are uncommon in Cervinae (Old World deer) and unknown in pronghorn (*Antilocapra americana*). Vestigial fifth metatarsals are common in the Bovidae, Capreolinae, and Cervinae; they are absent in giraffe (*Giraffa camelopardalis*) but present in about 40% of okapi (*Okapia johnstoni*) and pronghorn specimens (Silvia, Hamilton & Silvia, 2014).

In *Equus* the first and fifth metatarsals are lost, as are the phalanges of all but the third toe. The second and fourth metatarsals are vestigial (Figs. 12Y and 12Z). They remain as narrow splints that taper to a point distally without reaching the distal end of the third metatarsal (Nickel et al., 1986).

In the Rhinocerotidae (rhinos) and Tapiridae (tapirs) the first toe is lost and the first metatarsal is vestigial. In tapirs the first metatarsal is a small, blocky bone, almost cube-shaped in some specimens, at the tip of the ectocuneiform (the tarsal bone proximal to it). It is medially displaced onto the palmar surface of the foot (Fig. 12Z’). In rhinos the first metatarsal is a tiny nub that is fused to the end of the ectocuneiform (Radinsky, 1963).

The entire foot is lost in the Sirenia and Cetacea. For other extant mammal taxa, the phylogenetic distribution of character states (Fig. 1) indicates that the first toe became vestigial independently at least five times: in the Dasyuridae, Thryonomyidae, Canidae, Hyaenidae, and Rhinocerotidae + Tapiridae. It was independently lost eight times in mammals other than rodents: in *Chaeropus*, *Thylacinus*, Hyracoidea, Macroscelidea, sloths, Lagomorpha, Felidae, and Perissodactyla + Cetartiodactyla. Uncertainty in rodent phylogeny makes it difficult to tell how many times the fist toe was lost in rodents. If *Dipodomys* and *Zapus* are not sister genera, and if Chinchillidae and Caviidae + Dasyproctidae are not sister clades, then the toe was lost five times. If those two pairs of possible sister taxa are indeed pairs of sister taxa, then the toe was lost independently at least three times in the Rodentia. The second toe became vestigial independently at least twice: in *Chaeropus* and the Cervidae. It was lost independently four times: in the Camelidae, Antilocapridae, Giraffidae, and Bovidae. The third toe is vestigial in only one Recent mammalian taxon: *Chaeropus*. Among extant mammals, the fourth toe has been lost only in *Equus* and is not vestigial in any taxon. The fifth toe became vestigial at least twice: in *Chaeropus* and the Cervidae. It was independently lost ten times: in the Hyracoidea, sloths, Caviidae + Dasyproctidae, *Lagostomus*, Perissodactyla, Camelidae, Tayassuidae, Antilocapridae, Giraffidae, and Bovidae.

## Discussion

The pattern of vestigialization and loss in the forelimb does not match that of the hindlimb in any mammal taxon (Fig. 1). In nearly all taxa there is a lack of one-to-one correspondence in element reduction between fore and hind limbs (e.g., reduction of digit five both in the hand and in the foot). In addition, in nearly all taxa there is a lack of broader correspondence between element reduction between the two sets of limbs; that is, either the reduced/lost elements in one set of limbs do not correspond to the reduced/lost elements in the other set of limbs, or reduction/loss occurs in only one set of limbs but not the other. An exception to this rule is found in Camelidae, and Equidae, in the extant members of which the pattern of reduction/loss in the hand matches that of the foot; however, even in these taxa the pattern does not perfectly match between the forearm and the crus, because the fibula is vestigial but the ulna and radius are retained for their full lengths.

Among vertebrates, mammals are not exceptional in their lack of correlation in reduction/loss between the fore forelimb and hindlimb. In the Squamata, reduction and loss often occur in the forelimb before they occur in the hindlimb (Fürbringer, 1870; Essex, 1927; Kearney, 2002; Moch & Senter, 2011), although a few species exhibit the reverse pattern (Miralles et al., 2012). Both situations show a lack of correlation between forelimb and hindlimb. Dinosaurs also lack such correlation (Senter, 2010), and amphibians generally have a different number of digits on the hand than on the foot (Arnold, 2002). This suggests that in tetrapods generally, the genetic processes that result in reduction and loss in the forelimb are not connected with those that govern reduction and loss in the hindlimb.

Locomotor changes may have provided the selective pressure that drove the evolution of vestigiality in many mammalian skeletal structures. The limbs of therian mammals are upright, unlike the laterally sprawling limbs of their Mesozoic forebears and of monotremes (Kielan-Jaworowska & Hurum, 2006). Upright posture causes the middle digits to support most of the weight, rendering the outer digits expendable unless they are used for prehension. As a result of this expendability, mutations that reduce or delete such digits are not necessarily harmful, because they do not necessarily compromise limb functionality. For example, limb functionality does not seem to have been compromised by the reduction and loss of outer digits in hyraxes, hoofed mammals, canids, felids, and numerous rodent groups. Furthermore, in cursorial mammals, habitually fast locomotion provided selective pressure to reduce or lose side digits, because such reduction or loss lightens the foot, which is conducive to speed (Kardong, 2012). In the Sirenia and Cetacea, a switch from limb-driven locomotion to locomotion driven by dorsoventral tail undulation provided the selective pressure to reduce the hindlimb and pelvic girdle (Thewissen et al., 2009). Locomotor changes have driven vestigiality in limb and girdle structures in other taxa also. A shift from sprawling to parasagittal gait in the ancestors of dinosaurs engendered multiple parallel reductions and losses of dinosaurian outer digits (Senter, 2010). Vestigiality of limbs and girdles in various lizard taxa evolved after a shift from terrestrial, quadrupedal locomotion to subterranean burrowing or to laterally undulatory “grass-swimming” (Wiens & Slingluff, 2001).

It is interesting that in the Odontoceti, the vestigial pelvic girdle appears to retain only the ischium. With the exception of the burrowing snake genus *Typhlops* (Essex, 1927; List, 1966), in vestigial pelvic girdles of reptiles the ilium is usually the last bone to be lost (Moch & Senter, 2011). This suggests that the genetic processes governing pelvic girdle vestigialization are different between the Reptilia and Cetacea.

It is also interesting that in the reduced digits of the Pecora, the proximal elements (including the metapodials) are more reduced than the distal elements and are lost before the distal elements. In other mammalian taxa distal elements are reduced and lost before proximal elements. The latter is the norm in tetrapods generally (Essex, 1927; Stokely, 1947; Senter, 2010), with the exception of the vestigialization of the shaft of the first metatarsal of theropods (Senter, 2010). This suggests that the genetic processes governing digit reduction and loss are different in the Pecora than in other animals.

Anti-evolution authors often claim that vestigial structures do not exist, and some note that the lists of vestigial structures in biology textbooks have gotten smaller through the decades (Morris, 1974; Bergman & Howe, 1990; Bergman, 2000). They interpret this as a loss of confidence, by mainstream science, in the existence of vestigial structures. A recent survey of twenty-first-century primary scientific articles revealed the opposite: that the number of biological structures that scientists currently consider to be vestigial is enormous (Senter et al., 2015). In fact, new examples of previously-undescribed vestigial biological structures continue to be documented even in this century (Sekiguchi et al., 2002; Maslakova, Martindale & Norenburg, 2004; Tamatsu et al., 2007; Moch & Senter, 2011; Miralles et al., 2012). Unfortunately, however, the anti-evolution view that scientists have lost confidence in the existence of vestigial structures is reinforced by the shortness of the lists of only one to three examples of vestigial structures in recent biology textbooks (e.g., Starr & Taggart, 2004; Reece et al., 2011), including textbooks for evolution classes (e.g., Ridley, 2004; Kardong, 2008). There is even one textbook on evolution (Volpe & Rosenbaum, 2000) that does not mention vestigial structures at all. As shown here, mammals provide a plethora of examples of vestigial structures. Addition of these to lists of vestigial structures in textbooks and other media could prove helpful in countering the rejection of macroevolution that is prevalent in the United States and many European countries (Mazur, 2005; Miller, Scott & Okamoto, 2006). We therefore recommend that a wider variety of vestigial skeletal structures in mammals be included in such lists to counter young-Earth creationist claims and increase public acceptance of macroevolution. Toward this end, the numerous vestigial structures in dinosaurs (Senter, 2010), lizards (Fürbringer, 1870; Essex, 1927; Stokely, 1947; Kearney, 2002; Moch & Senter, 2011), invertebrates (e.g., Emerson, 1961; Gibert et al., 2000; Napoleão et al., 2005; Bowsher, 2007; Gotoh, Ito & Billen, 2013), plants (Wilson, 1982), and other organisms (Senter et al., 2015)—including the recently-discovered variety of vestigial organelles and endosymbionts in single-celled eukaryotes (Ludwig & Gibbs, 1989; Triemer & Lewandowski, 1994; Sato, Tews & Wilson, 2000; Sekiguchi et al., 2002; Regoes et al., 2005)—could also be cited. However, most people are more familiar with mammals and bones than with invertebrates and microbes and their anatomical structures. Citation of vestigial structures in mammal skeletons could therefore be of particular value due to the special impact conferred by familiarity.

## Institutional abbreviations

AKM: Alaska Museum of Natural History, Anchorage, Alaska, USA
AMNH: American Museum of Natural History, New York City, New York, USA
CM: Carnegie Museum of Natural History, Pittsburgh, Pennsylvania, USA
CTR: Carolina Tiger Rescue, Pittsboro, North Carolina, USA
FSU: Fayetteville State University, Fayetteville, North Carolina, USA
NAN: No accession number visible (specimens are mounted and on public display in most cases; FSU specimens are not on public display)
NCSM: North Carolina State Museum of Natural Sciences, Raleigh, North Carolina, USA
USNM: United States National Museum of Natural History, Washington, D.C., USA
YPM: Yale Peabody Museum, New Haven, Connecticut, USA

## Anatomical abbreviations

a: acromion process of scapula
ac: acetabulum
c: carpals
cl: clavicle
f: femur
fi: fibula
h: humerus
il: ilium
is: ischium
m: manubrium
mc: metacarpals
mt: metatarsals
of: obturator foramen
p: pubis
ph: phalanges
r: radius
s: sacrum
sc: scapula
st: sternebrae
t: tibia
ta: tarsals
u: ulna
I–V: first through fifth digits.

## Additional Information and Declarations

### Data Availability

The following information was supplied regarding data availability: All raw data are contained in the manuscript.
